# Essential phospholipids decrease apoptosis and increase membrane transport in human hepatocyte cell lines

**DOI:** 10.1186/s12944-022-01698-8

**Published:** 2022-09-24

**Authors:** Dominik Wupperfeld, Gert Fricker, Béatrice Bois De Fer, Larissa Frank, Annika Wehrle, Branko Popovic

**Affiliations:** 1 Institute of Pharmacy and Molecular Biotechnology, Ruprecht-Karls University of Heidelberg, Heidelberg, Germany; 2 Sanofi, Gentilly, France; 3 Division of Cellular Immunology, German Cancer Research Center (DKFZ), Heidelberg, Germany; 4 Sanofi, Industriepark Hoechst, Frankfurt am Main, Germany

**Keywords:** HepG2, HepaRG, Steatotic HepaRG, Nonalcoholic fatty liver disease

## Abstract

**Background:**

Essential phospholipids (EPL) have hepatoprotective effects across many liver diseases/conditions. The impact of EPL on hepatocyte function in vitro was investigated.

**Methods:**

Effects of noncytotoxic concentrations of EPL (0.1 and 0.25 mg/ml), and its constituents, polyenylphosphatidylcholine (PPC) and phosphatidylinositol (PI) (both at 0.1 and 1 mg/ml), on membrane fluidity, apoptosis and extracellular transport versus controls were investigated in human hepatocyte cell lines (HepG2, HepaRG, steatotic HepaRG).

**Results:**

Significantly increased membrane fluidity occurred with all 3 phospholipids (PLs) in HepG2 cultures, and with PI (1 mg/ml) in steatotic HepaRG cells. Significantly decreased tamoxifen-induced apoptosis was observed in HepG2 cells with EPL, PPC and PI. Breast cancer resistance protein (BCRP) activity was significantly increased by EPL and PI in HepG2 cells. Multidrug resistance-associated protein 2 (MRP-2) activity was unaffected by any PL in HepG2 cells, and significantly increased by EPL, PI and PPC (1 mg/ml) in HepaRG cells, and by PI (1 mg/ml) in steatotic HepaRG cells. Bile salt export protein (BSEP) activity in HepG2 cells and steatotic HepaRG cells was significantly increased by EPL (0.25 mg/ml), and PPC (both concentrations), but not by PI. The PLs had no effects on HepaRG cell BSEP activity. P-glycoprotein (P-GP) activity was significantly increased by all compounds in HepG2 cells. PI (1 mg/ml) significantly increased P-GP activity in HepaRG and steatotic HepaRG cells.

**Conclusions:**

EPL, PPC, and PI increased hepatocyte membrane fluidity, decreased apoptosis and increased hepatocellular export, all of which may improve liver function. These in-vitro investigations provide valuable insights into the mechanism of action of EPL.

**Supplementary Information:**

The online version contains supplementary material available at 10.1186/s12944-022-01698-8.

## Background

Chronic liver diseases are a major world public health problem. One worldwide estimate shows that 844 million people have chronic liver disease, with a mortality rate of 2 million deaths per year [1]. Nonalcoholic fatty liver disease (NAFLD) ranks among the most common chronic liver disease worldwide [2]; the disease spectrum ranges from simple fatty liver to nonalcoholic steatohepatitis (NASH), to fatty liver cirrhosis and hepatocellular carcinoma [2, 3]. The prevalence of NAFLD is particularly high in people with obesity (50%–90%) [4]. There are currently no FDA-approved drugs for treating NAFLD/NASH, and the mainstay of treatment is lifestyle changes such as weight loss and physical exercise [5–7]. Several types of medication [8] and herbal preparations [9] including essential phospholipids (EPL) [10, 11] are being evaluated and used as adjunctive treatment in NAFLD/NASH.

Of the treatments studied for use in NAFLD, EPL has the most evidence of an hepatoprotective effect [10–14], and is recommended in Russian [15] and Chinese [16] NAFLD guidelines. EPL preparations contain several phospholipids (PLs), including polyenylphosphatidylcholine (PPC; main component) and phosphatidylinositol (PI) [17]. PLs are essential components of mammalian cells. For instance, phosphatidylcholines are very common with key roles in several aspects of cellular function [12], and phosphoinositides have a major role in cell polarity [18].

Some clinical evidence exists to support the adjunctive use of EPL in liver diseases. For example, meta-analyses of clinical trials with EPL in patients with NAFLD have demonstrated significant reductions in circulating liver enzymes and lipid profiles, together with significant increases in the rate of overall improvement in steatosis and the likelihood of having no disease, and reduced likelihood of having moderate disease when used in combination with antidiabetic therapy [10]. In a range of alcoholic liver diseases, EPL resulted in several benefits compared with controls, e.g., significant decrease in mortality, higher response rates, and prevention of worsening of liver histopathology [13]. In patients with chronic viral hepatitis treated with adjunctive EPL treatment, a range of clinical benefits were reported including significant improvement in liver histology, greater reduction in liver enzymes, improved responses (e.g., fibrosis score) to viral hepatitis treatment, and improvement in subjective symptoms compared with controls [12]. EPL has also been used to treat or prevent chemical- or drug-induced liver injury, such as that seen with carbon tetrachloride, cyclosporin A, nonsteroidal anti-inflammatory drugs and anti-tuberculotic drugs [12].

Essentiale®, an EPL preparation, has been in use since 1957 (date of first marketing in Italy), and > 3.5 million units were prescribed between 2001–2013 (principally, oral capsules at 300 mg dose). The lipid profile of Essentiale® (mean, standard deviation [SD], mol/%) consists of phosphatidylcholine (61.94 [2.23]), lysophosphatidylcholine (16.18 [1.33]), phosphatidylethanolamine (PE) (4.85 [0.48]), PI (0.47 [0.22]), phosphatidylglycerol (0.31 [0.04]), phosphatidic acid (0.40 [0.06]), diacylglycerol (1.31 [0.38]) and triacylglycerol (13.50 [2.40]) [17]. Preclinical studies have provided insights into the multiple modes of action of EPL potentially involved in the hepatoprotective effects of such a formulation. The hepatocyte is a complex cell with many different functions presenting many potential targets for EPL [19], and EPL has a well-established mode of action. EPL influence membrane-dependent cellular functions and demonstrate anti-inflammatory, anti-steatotic, antioxidant, antifibrogenic, antiapoptotic, membrane-protective and lipid-regulating effects [12–14]. However, few data at the human hepatocyte cellular level have been reported.

Several of the potential mechanisms of EPL in preclinical studies such as disruption of membrane fluidity [20], apoptosis (i.e., programmed cell death) [21, 22], and hepatocyte transport proteins [19] are amenable to further study in vitro. A marker of apoptosis is caspase activity. This family of endoproteases have a critical role in regulating cell death and inflammation, and caspase-3 and caspase-7 are 2 executioner caspases involved in apoptosis [23]. Another potential therapeutic target in liver disease is transmembrane transport. Hepatocytes express numerous transmembrane transport proteins whose function strongly depends on membrane integrity, and many of these transport proteins are altered in liver diseases [24]. Several hepatocellular export proteins are involved in bile secretion, including breast cancer resistance protein (BCRP; biliary excretion), multidrug resistance-associated protein 2 (MRP-2; export of organic anions), bile salt export protein (BSEP; bile salt export), and P-glycoprotein (P-GP; export of xenobiotics and endogenous metabolites) [24]. Transporters of bile acids are potentially involved in a wide range of liver disorders [25–27].

Primary human hepatocytes are the gold standard for in-vitro evaluation of cellular mechanisms, but are not readily available [28]. Thus, immortal hepatic cell lines are utilized [28]. HepG2 cells are widely used as a model for human hepatic cell functions [29]. HepaRG cells are another human hepatic cell line that is suited for investigating glucose and fatty acid metabolism, perturbation of which is linked to metabolic liver diseases [30]. Steatosis can be induced in HepaRG cells as another model to evaluate fatty liver disease [31], giving rise to an evaluable steatotic HepaRG cell line. However, it is known that HepG2 and HepaRG cells have differences in drug-metabolizing enzymes, drug transporters, and gene expression profiles [32, 33]. To strengthen the understanding of the clinically observed hepatoprotective properties of lipids containing unsaturated fats, the effects of EPL (Essentiale®), and its constituents PPC and PI on hepatic cellular function in human cell lines (HepG2 cells, HepaRG cells, steatotic HepaRG cells) were investigated. The functions evaluated and reported herein were: membrane fluidity, apoptosis (programmed cell death) and hepatocellular transport.

## Methods

### Chemicals and reagents

Dimethyl sulfoxide (DMSO), insulin, 1,6-diphenyl-1,3,5-hexatriene (DPH), stearic acid, oleic acid, tamoxifen, valspodar (PSC833), calcein-am, sulforhodamine 101, Ko143, MK571, and mitoxantrone were purchased from Sigma-Aldrich (St Louis, MO, USA). Hydrocortisone hemisuccinate was obtained from Santa Cruz Biotechnology (Dallas, TX, USA). Penicillin/streptomycin, fetal bovine serum (FBS) were purchased from Biochrom (Berlin, Germany). 7-β-NBD-taurocholate was custom-synthesized as described by Schneider et al. [34] PPC and PI were obtained from Lipoid (Ludwigshafen am Rhein, Germany). EPL (Essentiale Forte 300 mg) was obtained from Sanofi. All other chemicals were purchased from commercial sources and were of the highest purity available.

### Preparation of liposomes

To obtain sufficiently high concentrations of EPL, PPC and PI in aqueous cell culture media, these PLs were incorporated into liposomes. Liposomes were prepared by dual centrifugation (ZentriMix 380 R, Andreas Hettich GmbH &Co KG, Tuttlingen, Germany) as described previously [35]. The mass of PL used was set as 100%. SiLibeads (TypZY-P 1.4–1.6 mm, Art.No.: 9715–41, Sigmund Lindner GmbH, Warmensteinach, Germany) were added to the appropriate PL, at 1000% (vs. lipid) or a minimum of 1 g and cell culture medium was added to 150% of the PL mass used. The resulting mixture was subjected to dual centrifugation (15 min, 2340 rpm). Second and third centrifugation runs (3 min, 2340 rpm) were performed with additional cell culture medium volumes of 300% and 550%, respectively. The liposomal dispersion was then diluted to a PL concentration of 50 mg/ml and filtered through mixed cellulose esters membrane (0.45 µm pore size; Merck KGaA, Darmstadt, Germany).

### Cell lines and culture conditions

The HepG2 cell line was purchased from Sigma-Aldrich (Acc No: 85011430, Lot: 16K046; St. Louis, MO, USA). For all experiments, HepG2 cells were seeded at a density of 1.4 × 10^5^ cells/cm^2^. This cell line was routinely cultured in Roswell Park Memorial Institute 1640 medium supplemented with 10% FBS, 100 U/ml penicillin, and 100 µg/ml streptomycin.

Fully differentiated HepaRG cells, obtained from Lonza (Cat. No: NSHPRG, Walkersville, MD, USA), were cultured as previously described by Le Guillou et al. [36]. Briefly, differentiated HepaRG cells were seeded at a density of 2.25 × 10^5^ cells/cm^2^ and incubated in Williams’ medium E supplemented with 5% FBS, 100 U/ml penicillin, 100 µg/ml streptomycin, 2 mM glutamine, 5 µg/ml insulin, 50 µM hydrocortisone hemisuccinate, and 1% DMSO.

To induce steatosis, HepaRG cells were treated for a 2 week-period with a mixture of stearic acid (150 µM) and oleic acid (150 µM). Stearic acid and oleic acid were dissolved in DMSO; the final concentration of DMSO was always maintained at 1% in the cultures.

All cell lines were cultured at 37 °C in an atmosphere of 5% CO_2_ and 95% humidity. Cell culture medium was renewed every 2 or 3 days.

### Evaluation of cytotoxicity

To enable selection of 2 noncytotoxic concentrations of each PL preparation for evaluation of their impact on hepatocyte apoptosis and transport function, cytotoxicity of all compounds was evaluated in HepG2 and HepaRG cell lines using PrestoBlue® cell viability reagent (Cat. No: A13261, Thermo Fisher, Waltham, MA, USA). Living cells reduce the resazurin in the solution to resorufin, which is red in colour and highly fluorescent. The colour change is detected using fluorescence measurements and correlates with cell viability [37].

The cell lines, HepG2 and HepaRG, were seeded and cultured as described above. The PLs of interest were added to these cultures via liposome preparations (see above) and the cultures were incubated for 48 h. The concentrations of PL preparations evaluated for cytotoxicity in HepG2 cells were: EPL: 0, 0.0075, 0.0375, 0.075, 0.375, 0.75, 3.75, 7.5 and 37.5 mg/ml; PPC: 0, 0.009, 0.045, 0.09, 0.45, 0.9, 4.5, 8.9 and 44.5 mg/ml; and PI: 0, 0.0073, 0.036, 0.073, 0.36, 0.73, 3.6, 7.3 and 36.3 mg/ml. For the HepaRG cell line, all PL preparations were evaluated at 0.01, 0.02, 0.1, 0.2, 1, 2, 10 and 20 mg/ml. Following incubation, a PrestoBlue cell viability assay was performed according to the manufacturer’s instructions. For this assay, the cell viability reagent was diluted in the culture medium. After incubation for 30 min at 37 °C in a cell culture incubator the fluorescence intensities (excitation: 540/25 nm; emission: 590/20 nm) was detected using a Tecan Fluoroscan infinite F200 pro plate reader (Tecan, Männedorf, Switzerland).

### Detection of membrane fluidity using fluorescence anisotropy

As a PL bilayer membrane, the plasma cell membrane is anisotropic, in which lipid resistance to motion is different in different directions; rapid motions include lateral diffusion and rotational diffusion, whereas transverse diffusion is very slow [38]. Molecular interactions within the cell membrane are complex and membrane fluidity impacts these interactions and subsequent functions [38, 39]. Dietary fats have been shown to impact membrane fluidity [40]. Membrane fluidity assessed by anisotropy uses the fluorescent probe, DPH; a decrease in anisotropy signifies an increase in membrane fluidity [41].

This technique was used by Dudeja et al. [42] to determine membrane fluidity of cells [42]. In the present evaluations, the method of Dudeja was used, with the exception that DPH was used, which is similar to 1-(4-trimethylammoniumphenyl)-DPH [41]. HepG2, HepaRG and steatotic HepaRG cells in culture were incubated with EPL (0.1 mg/ml and 0.25 mg/ml), PPC (0.1 mg/ml and 1 mg/ml) or PI (0.1 mg/ml and 1 mg/ml) for 48 h. The pre-treated cells were then incubated with 10 µM DPH for 4 h at 37 °C. Fluorescence intensities (excitation: 360/25 nm; emission: 430/25 nm) with different polarization levels were detected using a Tecan Fluoroscan infinite F200 pro plate reader (Tecan, Männedorf, Switzerland). Negative fluorescence anisotropy values are due to the predefined distinct g-factor of the instrument. Relative differences between values of different cell lines and treatments are not affected by this factor.

### Analysis of cell apoptosis

Caspase-3 and caspase-7 are involved in apoptosis [23]. The mechanism of hepatotoxicity induced by tamoxifen involves induction of apoptosis [43, 44]. Thus, the activity of caspases 3 and 7 are appropriate markers of apoptosis in vitro, with tamoxifen as a positive control. CellEvent™ caspase-3/-7 green flow cytometry kit (Cat. No: C10427, Thermo Fisher, Waltham, MA, USA) contains a novel fluorogenic substrate consisting of a four-amino acid peptide conjugated to a nucleic acid-binding dye which is nonfluorescent. The peptide sequence is a cleavage site for activated caspase-3/-7. Apoptosis is detected in live cells with this reagent, as caspase-3/-7 is activated and cleaves the peptide sequence enabling the dye to bind to DNA resulting in a bright fluorescent response.

The cell lines, HepG2, HepaRG and steatotic HepaRG, were seeded and cultured as described above. The PLs were added (EPL: 0.1 mg/ml and 0.25 mg/ml; PPC: 0.1 mg/ml and 1 mg/ml; PI: 0.1 mg/ml and 1 mg/ml) to these cultures via liposome preparations (see above) to detect if they had an effect on apoptosis induction in these cells. The cultures were incubated with PLs for 48 h. To induce apoptosis, the cell lines in culture were treated with tamoxifen, in the absence and presence of PL preparations. Preliminary tests showed that HepaRG cells needed slightly higher tamoxifen concentrations to show a caspase-3/7 fluorescence signal compared with HepG2 cells (data not shown). Thus, HepG2 cells were incubated for 3 h with either 42 µM or 55 µM tamoxifen, and HepaRG and steatotic HepaRG were incubated for 4 h with 45 µM or 60 µM tamoxifen.

To evaluate the induction of apoptosis, treated cells were harvested and resuspended at a cell concentration of 1 × 10^6^ cells/ml in a fluorescence-activated cell sorting (FACS) buffer (phosphate buffered saline [PBS] supplemented with 5% FBS) and a concentration of 500 nM caspase-3/-7 green detection reagent. The samples were then incubated for 30 min at 37 °C in an atmosphere of 5% CO_2_. During the final 5 min of this incubation, SYTOX™ AADvanced™ solution was added at a final concentration of 1 µM. Cells were then stored on ice until analysis by flow cytometry on a FACS Fortessa LSRII (Becton Dickinson) using a 488 nm excitation and collecting fluorescence emission with a 530/30 optical filter (FITC channel) for caspase-3/-7 green detection reagent and a 695/40 optical filter (PerCP-Cy5.5 channel) for SYTOX™ AADvanced™. Cells were pre-gated for size and exclusion of doublets by forward scatter and side scatter (Supplementary Fig. S1). Dead cells were defined as SytoxAAD-positive cells and SytoxAAD-negative cells were further analysed for caspase-3/-7 staining as a marker for induction of apoptosis. Data were analysed using FlowJo software (TreeStar Inc).

### Analyses of hepatocyte transport function

HepG2, HepaRG and steatotic HepaRG cells were seeded and cultured as described above. The PLs were added (EPL: 0.1 mg/ml and 0.25 mg/ml; PPC: 0.1 mg/ml and 1 mg/ml; PI: 0.1 mg/ml and 1 mg/ml) to these cultures via liposome preparations (see above) to detect if they had an effect on hepatocyte transport function in these cells. The cultures were incubated with PLs for 48 h. For the detection of transporter activity, the cells were treated with a model fluorogenic substrate specific to each of the transporters evaluated [34, 45–47].

For BCRP, MRP-2 and P-GP assays, the PL pre-treated cells were incubated with the appropriate model substrate at 37 °C for a specified time in the dark; BCRP: 20 µM mitoxantrone, 3 h; MRP-2: 1 µM sulforhodamine 101, 60 min on a temperature-controlled shaker; P-GP: 1 µM calcein-am, 30 min on a temperature-controlled shaker. These incubation times and substrate/inhibitor concentrations were selected following preliminary studies, conducted to determine which combinations resulted in the highest difference in fluorescence signal. Inhibitors of these transport proteins were included as positive controls. PL pre-treated cells were incubated with appropriate inhibitor; i.e., 20 µM KO143 for BCRP inhibition for 1 h before mitoxantrone addition 20 µM (HepG2 cells) or 50 µM (HepaRG and steatotic HepaRG cells) MK571 for MRP-2 inhibition for 30 min before sulforhodamine 101 addition; and 1 µM PSC833 for P-GP inhibition for 15 min before calcein-am addition. The cells were then washed twice with ice-cold PBS to remove the fluorescent molecules on the outside of the cells. The washed cells were lysed by adding pre-warmed 1% (v/v) Triton X-100 and incubating for 30 min at 37 °C. Fluorescence was measured using Tecan Fluoroscan Infinite 200 PRO plate reader (Tecan, Männedorf, Switzerland). The excitation/emission wavelengths used were: BCRP 615 nm/670 nm; MRP-2 540 nm/ 590 nm; P-GP 485 nm/535 nm.

Evaluation of BSEP activity was conducted as previously described [46]. HepG2, HepaRG and steatotic HepaRG cells were cultured in chamber slides (Cat. No: C7182, Sigma-Aldrich, St Louis, MO, USA) with the appropriate concentrations of EPL, PPC or PI for 48 h. The fluorescent bile acid, 7-beta-NBD-taurocholate (10 µM) was added to the cells and incubated for 1 h at 37 °C in the dark. The cells were then washed twice with ice-cold PBS to remove the extracellular fluorescent dye. To visualize the fluorescent bile acid in the canaliculi structures, the slides were placed on the stage of a Leica inverted laser scanning microscope (TCS SP5II) and its program LAS AF and viewed with a 40 × water immersion objective. The excitation for 7-beta-NBD-taurocholate confocal fluorescent images was provided by a 488 nm-laser line and an emission at 550/25 nm. Photomultiplier gain was adjusted for each chamber slide individually to ensure that cells autofluorescence and background fluorescence were undetectable. The plane was adjusted so that the canaliculi structures were sharply focused, and final images were acquired using the line-average of 2 and frame-accumulation of 4 at 2048 × 2048 pixels by 8 bits. Afterwards, the mean fluorescence intensities of canaliculi structures were determined using the program ImageJ software version 1.52p.

### Ethics

All authors had access to the study data, reviewed drafts of the manuscript and provided approval of the final manuscript. The study was conducted in line with International Council for Harmonisation Good Laboratory Practice.

### Statistical analyses

No formal sample size was determined. All data were analysed using SAS® software version 9.4 or higher (SAS institute Inc. Cary NC USA). Descriptive statistics were performed (n, arithmetic mean, and standard deviation) for each parameter. Data were displayed graphically.

Each experiment was conducted between 1 and 4 times, within each experiment 2 to 6 replicates per treatment were performed. The mean values for the replicates in each experiment were calculated, then the mean values across the experiments were calculated from the means of the replicates. For each cell line evaluated and for each parameter assessed, comparisons were made between each treatment group (EPL, PPC, PI at different concentrations) versus untreated controls (non-PL treated cells). No statistical comparisons were made between EPL, PPC and PI or between cell lines.

Cytotoxicity was not statistically analysed. For anisotropy, BCRP, MRP-2, BSEP and P-GP activity, comparisons were made using analysis of variance (ANOVA) including treatment group as a fixed factor and a Dunnett adjustment for pairwise comparisons. For caspase-3/-7 results, comparisons were made using ANOVA including treatment group, tamoxifen levels, and treatment group*tamoxifen level interaction as fixed factors and a Tukey adjustment for pairwise comparison. A threshold of 5% was used to denote statistical significance for all pairwise comparisons.

## Results

### Cytotoxicity of EPL, PPC and PI in HepG2 and HepaRG cell lines

Addition of EPL to HepG2 cell line cultures at concentrations ≥ 0.375 mg/ml resulted in a marked and concentration-dependent reduction in cell viability (Table 1). Cell viability in HepG2 cells ranged, on average, from 93.0%–102.6% (as a percentage of untreated controls) with PPC at concentrations ≤ 0.9 mg/ml; at higher concentrations there was a modest reduction in cell viability. For PI addition to HepG2 cell line cultures, cell viability ranged, on average, from 92.3%–93.3% (as a percentage of untreated controls) with concentrations ≤ 0.073 mg/ml; PI concentrations 0.36–7.3 mg/ml resulted in a modest decrease in cell viability, whereas PI at 36.3 mg/ml had a severe impact on cell viability (Table 1).

**Table 1:** Cytotoxicity of EPL, PPC and PI in the HepG2 cell line

Cell viability (% of untreated cells)	EPL concentration mg/ml								
	**0**	**0.0075**	**0.0375**	**0.075**	**0.375**	**0.75**	**3.75**	**7.5**	**37.5**
**Mean (SD) [range]**	97.25 (0.69) [96.7–98.0]	96.08 (10.87) [86.7–105.7]	97.08 (5.49) [92.3–102.0]	96.17 (5.39) [91.3–101.0]	71.75 (1.55) [70.3–73.7]	47.83 (0.88) [47.0–49.0]	23.42 (0.74) [22.7–24.3]	24.67 (0.98) [23.3–25.7]	4.08 (0.69) [3.3–4.7]
	**PPC concentration mg/ml**								
	**0**	**0.009**	**0.045**	**0.09**	**0.45**	**0.9**	**4.5**	**8.9**	**44.5**
**Mean (SD) [range]**	100.17 (1.04) [99.0–101.3]	102.58 (3.84) [98.3–107.0]	102.17 (4.24) [97.7–107.3]	99.50 (5.01) [95.0–106.7]	93.00 (3.14) [91.0–97.7]	90.08 (4.47) [86.0–95.7]	81.58 (4.13) [75.7–85.0]	76.67 (5.13) [70.3–81.0]	63.25 (9.87) [49.0–70.7]
	**PI concentration mg/ml**								
	**0**	**0.0073**	**0.036**	**0.073**	**0.36**	**0.73**	**3.6**	**7.3**	**36.3**
**Mean (SD) [range]**	100.17 (1.04) [99.0–101.3]	93.17 (8.46) [84.3–101.7]	93.33 (8.54) [86.3–105.7]	92.33 (6.32) [84.7–99.0]	84.08 (3.56) [79.7–88.3]	78.75 (3.40) [74.7–82.7]	75.17 (2.73) [72.7–79.0]	60.33 (6.31) [55.3–69.3]	3.00 (0.47) [2.7–3.7]

Values shown are mean ± SD (range) as percentage of untreated cells (no PL addition) for 4 separate experiments; *n* = 3 replicate wells for each concentration of each compound per experiment

*EPL* Essential phospholipids, *PI* Phosphatidylinositol, *PL* Phospholipid, *PPC* Polyenylphosphatidylcholine, *SD* Standard deviation

Mean fluorescence of resorufin in the culture medium ranged from 101.0%–123.0% (EPL), 95.9%–129.1% (PPC), and 98.8%–123.1% of control values (untreated HepaRG cells) following incubation for 48 h with EPL, PPC, or PI, respectively. Thus, none of the evaluated concentrations (0.01–20 mg/ml) of EPL, PPC and PI showed cytotoxicity in the HepaRG cell line in culture (Supplementary Table S1).

Based on these data, the concentrations of PLs selected for further evaluations in hepatocyte cell lines were 0.1 and 0.25 mg/ml EPL, 0.1 and 1 mg/ml PPC, and 0.1 and 1 mg/ml PI, as these concentrations were considered to be the highest concentrations without major cytotoxicity.

### Effect of EPL, PPC and PI on membrane fluidity in HepG2, HepaRG and steatotic HepaRG cell lines

**Fig. 1:**
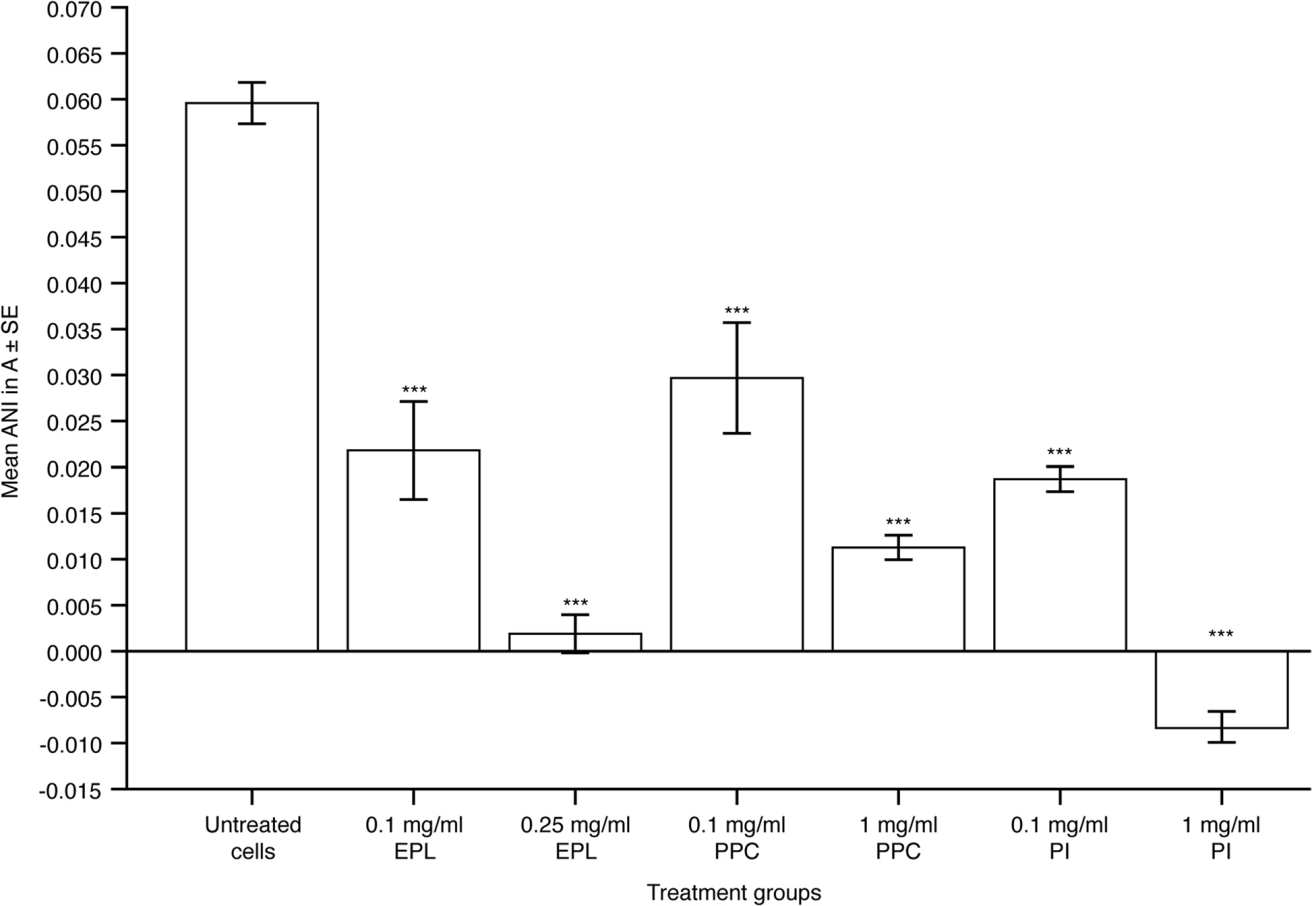
Effect of EPL, PPC and PI on anisotropy in the HepG2 cell line. Values shown are mean ± SE for 4 separate experiments; *n* = 1 well for each concentration of each compound per experiment. ***P < 0.001 versus untreated cells. ANI, anisotropy; EPL, essential phospholipids; PI, phosphatidylinositol; PPC, polyenylphosphatidylcholine; SE, standard error. Supplementary Table S2 shows the statistical analyses

Figure 1 and Supplementary Table S2 depict anisotropy measurements in HepG2 cells (decreased anisotropy values signify increased membrane fluidity). EPL addition resulted in a concentration-dependent and significant decrease in anisotropy measurements versus untreated cells; least-square (LS) mean differences (95% confidence intervals [CI]) versus untreated cells were –0.038 (–0.048 to –0.027; *P* < 0.001) and –0.058 (–0.041 to –0.047; *P* < 0.001) with 0.1 and 0.25 mg/ml EPL, respectively. PPC also significantly reduced anisotropy measurements, at both concentrations, in HepG2 cells; LS mean differences (95% CI) versus untreated cells were –0.030 (–0.041 to –0.019; *P* < 0.001, 0.1 mg/ml PPC) and –0.048 (–0.059 to –0.038; *P* < 0.001, 1 mg/ml PPC). Membrane fluidity (decreased anisotropy) was also significantly increased in HepG2 cells by incubation with PI; LS mean differences (95% CI) versus untreated cells in anisotropy values were –0.041 (–0.052 to –0.030; *P* < 0.001, 0.1 mg/ml PI) and –0.068 (–0.078 to –0.057; *P* < 0.001, 1 mg/ml PI). At 0.1 mg/ml, the largest decrease in anisotropy from untreated cells was seen with PI (68% decrease) and the lowest decrease was with PPC (50% decrease), and EPL resulted in a 63% decrease.

In HepaRG cells, the only significant effect on anisotropy was a decrease with 1 mg/ml PI versus untreated controls (LS mean difference [95% CI] –0.032 [–0.056 to –0.008], *P* < 0.01) (Supplementary Fig. S2A, Supplementary Table S2). EPL and PPC had no significant impact on membrane fluidity in HepaRG cells at the concentrations evaluated. In steatotic HepaRG cells, EPL and PPC did not affect membrane fluidity, whereas PI addition did significantly decrease anisotropy (LS mean difference [95% CI] –0.009 [–0.018 to –0.0004], *P* < 0.05) (Supplementary Fig. S2B, Supplementary Table S2).

### Analyses of cell apoptosis

#### HepG2 cells

**Fig. 2:**
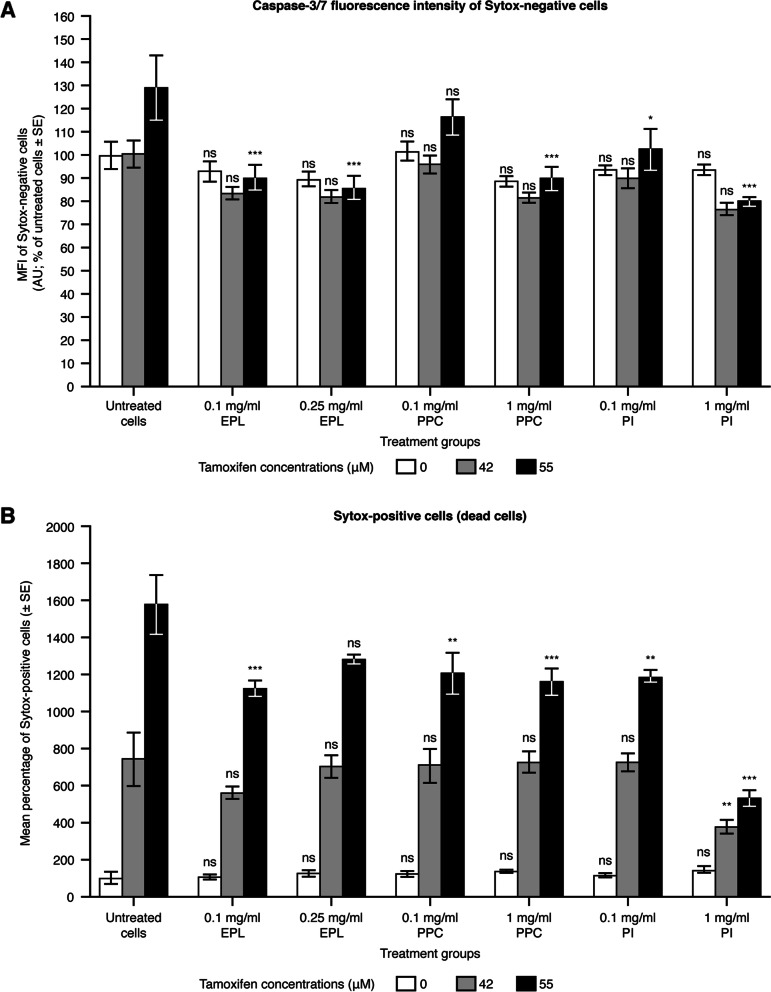
Effect of EPL, PPC and PI on apoptosis in the HepG2 cell line. Values shown are mean ± SE (as % of untreated cells) for 2 separate experiments; *n* = 2 wells for each concentration of each compound per experiment. ns: not significant, *P < 0.05, **P < 0.01, ***P < 0.001 versus untreated cells. Note: for untreated HepG2 cells, 1.43% of cells were found to be Sytox positive (dead cells). Here, these values are presented as percentages, as the results are normalized to untreated cells. AU, arbitrary units; EPL, essential phospholipids; ns, not significant; MFI, median fluorescence intensity; PI, phosphatidylinositol; PPC, polyenylphosphatidylcholine; SE, standard error. Supplementary Table S3 shows the statistical analyses

Figure 2A and Supplementary Table S3 show the effects of tamoxifen (positive control for apoptosis), EPL, PPC, and PI, and the combination of tamoxifen and each PL on apoptosis induction in HepG2 cells, as assessed by caspase-3/-7 fluorescence intensity of Sytox-negative cells. Tamoxifen resulted in a slight increase in caspase-3/-7 staining at the highest concentration (55 µM) only compared with untreated cells. The concentrations of EPL, PPC and PI evaluated did not induce apoptosis in HepG2 cells (i.e., in the absence of tamoxifen), as there were no significant differences in fluorescence intensity compared with untreated cells. EPL, PPC, or PI addition to HepG2 cells with 42 µM tamoxifen also had no impact on fluorescence staining compared with non-PL exposed cells exposed to 42 µM tamoxifen. EPL significantly reduced 55 µM tamoxifen-induced apoptosis at both concentrations evaluated, i.e., LS mean difference (95% CI) –38.8 (–63.4 to –14.1), *P* < 0.001 with 0.1 mg/ml EPL, and –43.2 (–67.8 to –18.51), *P* < 0.001 with 0.25 mg/ml EPL. PPC significantly reduced tamoxifen-induced apoptosis (55 µM) only at the highest concentration evaluated i.e., 1 mg/ml: LS mean difference (95% CI) –39.3 (–64.0 to –14.7), *P* < 0.001. PI addition to HepG2 cells significantly reduced tamoxifen-induced apoptosis (55 µM) at both concentrations evaluated; 0.1 mg/ml LS mean difference (95% CI) –26.5 (–51.1 to –1.8), *P* < 0.05; 1 mg/ml: –49.1 (–73.8 to –24.5), *P* < 0.001.

To confirm the effects of the PLs on tamoxifen-induced apoptosis, an evaluation was also conducted of Sytox-positive cells (Fig. 2B, Supplementary Table S3), as these cells are dead and should only increase in number as a result of apoptosis induction. The number of dead cells was normalized against Sytox-positive untreated cells (without PLs or tamoxifen) and the normalized percentage of dead Sytox-positive cells was compared to that in Sytox-negative cells. Tamoxifen produced a marked concentration-dependent increase in the percentage of dead cells in untreated HepG2 cells. In the absence of tamoxifen, EPL, PPC, and PI had no significant effect on Sytox-positive cells in HepG2 cells. Tamoxifen-induced Sytox-positive cells (42 µM) were not affected by either 0.1 or 0.25 mg/ml EPL, or by either 0.1 or 1 mg/ml PPC. In contrast, the percentage of tamoxifen-induced (42 µM) dead HepG2 cells was significantly decreased in the presence of 1 mg/ml PI (LS mean difference [95% CI] –364.0 [–663.0 to –64.9], *P* < 0.01), but not by the lower PI concentration of 0.1 mg/ml. In the presence of tamoxifen at 55 µM, EPL reduced the percentage of dead cells at both concentrations evaluated, which was significant for 0.1 mg/ml EPL (LS mean difference [95% CI] –452.8 [–751.8 to –153.8], *P* < 0.001) but not for 0.25 mg/ml EPL (LS mean difference [95% CI] –293.7 [–592.7 to –5.3], *P* = 0.0599). PPC significantly reduced tamoxifen-induced Sytox-positive cells (55 µM) at both PPC concentrations, i.e., 0.1 mg/ml (LS mean difference [95% CI] –372.4 [–671.4 to –73.4], *P* < 0.01), and 1 mg/ml (LS mean difference [95% CI] –416.1 [–715.1 to –117.1], *P* < 0.001). For tamoxifen at 55 µM, PI significantly reduced apoptosis at both 0.1 mg/ml (LS mean difference [95% CI] –388.1 [–687.2 to –89.1], *P* < 0.01), and 1 mg/ml (LS mean difference [95% CI] –1045.3 [–1334.3 to –746.2], *P* < 0.001).

#### HepaRG cells

Supplementary Fig. S3A and Supplementary Table S4 show the effects of tamoxifen and each PL on apoptosis induction in HepaRG cells (caspase-3/-7 fluorescence intensity of Sytox-negative cells). Tamoxifen addition to HepaRG cells increased caspase-3/-7 staining at both concentrations evaluated compared with untreated cells. In the absence of tamoxifen, none of the PLs induced apoptosis in HepaRG cells. EPL, PPC, and PI at the concentrations tested had no significant effect on caspase-3/-7 staining in presence of either tamoxifen concentration.

The effects of tamoxifen, EPL, PPC, and PI on the percentage of dead HepaRG cells are shown in Supplementary Fig. S3B and Supplementary Table S4. Tamoxifen induced a concentration-dependent increase in the percentage of dead cells. In the absence of tamoxifen, EPL and PPC had no significant effect on the percentage of dead HepaRG cells at the concentrations evaluated. Addition of PI to untreated HepaRG cells had no significant effect at 0.1 mg/ml and slightly increased the percentage of dead cells at 1 mg/ml (LS mean difference [95% CI] 85.7 [2.6–168.7], *P* < 0.05). EPL addition to HepaRG cells had no significant effects on tamoxifen-induced (45 µM) cell death. PPC addition to HepaRG cells at 0.1 mg/ml significantly increased the percentage of dead cells in the presence of 45 µM tamoxifen (LS mean difference [95% CI] 116.9 [33.8–199.9], *P* < 0.001); although PPC at 1 mg/ml had no effect. With PI, the percentage of dead cells in the presence of 45 µM tamoxifen (LS mean difference [95% CI] 90.0 [7.0–173.1], *P* < 0.05), whereas 1 mg/ml PI had no effect on this endpoint. With tamoxifen at 60 µM in HepaRG cells, neither EPL nor PI at the concentrations evaluated had any effect on the percentage of dead cells. PPC addition to HepaRG cells at 0.1 mg/ml significantly decreased the percentage of dead cells induced by 60 µM tamoxifen (LS mean difference [95% CI] 100.1 [33.8–199.9], *P* < 0.001); whereas 1 mg/ml PPC had no effect on this endpoint.

#### Steatotic HepaRG cells

Tamoxifen addition to steatotic HepaRG cells slightly increased apoptosis at both concentrations evaluated (Supplementary Fig. S4A, Supplementary Table S5). In this cell line, EPL, PPC, and PI at the concentrations tested had no significant effects on caspase-3/-7 staining in the absence or presence of tamoxifen (Supplementary Fig. S4A, Supplementary Table S5).

Tamoxifen slightly increased the percentage of dead HepaRG cells in untreated cells. This increase was concentration-dependent; overall tamoxifen levels effect *P* < 0.0001 (Supplementary Fig. 4B, Supplementary Table S5). In the absence of tamoxifen, EPL, PPC, and PI had no significant effect on the percentage of dead steatotic HepaRG cells. For both concentrations of tamoxifen used (45 and 60 µM), EPL, PPC, and PI had no significant effect on the percentage of dead cells at any concentration evaluated (Supplementary Fig. S4B, Supplementary Table S5).

### Analyses of hepatocyte transport function

#### BCRP

Intracellular accumulation of the model substrate, mitoxantrone, was used to assess the activity of BCRP in cell culture, i.e., decreased intracellular concentrations of mitoxantrone indicate increased activity of BCRP in the extracellular transport of this substrate. As a positive control, the BCRP inhibitor KO143 was added to each cell line in the absence of PL.

**Fig. 3:**
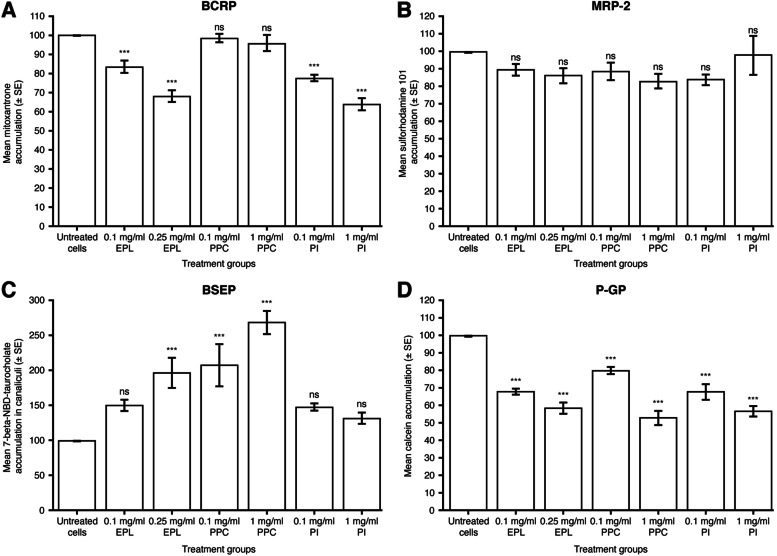
Effect of EPL, PPC and PI on hepatocellular transport protein activity in HepG2 cell line. Values shown are mean ± SE (cellular substrate accumulation as percentage of untreated cells) for 4 (MRP-2, BSEP, P-GP) or 5 (BCRP) experiments; *n* = 1 (BCRP), 4 (MRP-2, BSEP) or 6 (P-GP) wells for each concentration of each compound/experiment. ns: not significant; ***P < 0.001 versus untreated cells. BCRP, breast cancer resistance protein; BSEP, bile salt export protein; EPL, essential phospholipids; MRP-2, multidrug resistance-associated protein 2; P-GP, P-glycoprotein; PI, phosphatidylinositol; PPC, polyenylphosphatidylcholine; SE, standard error. Supplementary Table S6 shows the statistical analyses

As a percentage of untreated HepG2 cells, the mean (SD) intracellular concentration of mitoxantrone was 123.4 (7.33; *n* = 5 replicate wells in 1 experiment) in the presence of KO143, indicating BCRP inhibition. In HepG2 cell cultures, compared with untreated controls, EPL statistically significantly decreased mitoxantrone accumulation at both 0.1 mg/ml (LS mean difference [95% CI] –16.2 [–26.0 to –6.4], *P* < 0.001) and 0.25 mg/ml (LS mean difference [95% CI] –31.8 [–41.6 to –22.0], *P* < 0.001) (Fig. 3A, Supplementary Table S6). PI also statistically significantly decreased mitoxantrone accumulation at both 0.1 mg/ml (LS mean difference [95% CI] –22.2 [–32.0 to –12.4], *P* < 0.001) and 1 mg/ml (LS mean difference [95% CI] –36.0 [–45.8 to –26.2], *P* < 0.001) versus untreated controls, whereas PPC addition to HepG2 cells had no significant effect on the BCRP activity at either concentration tested (Fig. 3A, Supplementary Table S6).

As a percentage of untreated cells, the mean (SD) intracellular concentrations of mitoxantrone were 111.4 (7.05; *n* = 4 experiments) and 117.4 (3.44; *n* = 4 experiments) in HepaRG and steatotic HepaRG cells, respectively, in the presence of KO143, indicating BCRP inhibition. Addition of EPL, PPC, or PI had no significant effects on BCRP activity in vitro in either HepaRG cells (Supplementary Fig. S5A, Supplementary Table S7) or steatotic HepaRG cells (Supplementary Fig. S6A, Supplementary Table S8).

#### MRP-2

For assessing MRP-2 activity in vitro, the model substrate used was sulforhodamine 101. Decreased intracellular concentrations of this substrate indicate increased activity of MRP-2 in extracellular transport. As a positive control, the MRP-2 inhibitor MK571 was added to each cell line in the absence of PL.

As a percentage of untreated HepG2 cells, the mean (SD) intracellular concentration of sulforhodamine 101 was 168.9 (28.54; *n* = 4 experiments) in the presence of MK571, indicating MRP-2 inhibition. There was no significant effect of EPL, PPC, or PI addition to HepG2 cell cultures on sulforhodamine 101 accumulation at the concentrations evaluated versus untreated controls (Fig. 3B, Supplementary Table S6).

The mean (SD) intracellular concentration of sulforhodamine 101 was 141.0% (5.5; *n* = 4 experiments) of untreated HepaRG controls, in the presence of MK571, indicating MRP-2 inhibition. In HepaRG cells, intracellular accumulation of sulforhodamine 101 was significantly reduced by EPL addition at both 0.1 mg/ml (LS mean difference [95% CI] –10.4 [–19.1 to –1.7], *P* < 0.05) and 0.25 mg/ml (LS mean difference [95% CI] –12.9 [–21.6 to –4.2], *P* < 0.01) versus untreated cells (Supplementary Fig. S5B, Supplementary Table S7). MRP-2 activity in HepaRG cells was significantly increased by the addition of 1 mg/ml PPC (LS mean difference [95% CI] –14.9 [–23.6 to –6.2], *P* < 0.001) versus untreated cells, whereas the 0.1 mg/ml concentration had no significant effect. PI addition to HepaRG cells also significantly increased MRP-2 activity in vitro at 0.1 mg/ml (LS mean difference [95% CI] –9.6 [–18.3 to –0.9], *P* < 0.05) and 1 mg/ml (LS mean difference [95% CI] –22.3 [–31.0 to –13.5], *P* < 0.001) versus untreated cells (Supplementary Fig. S5B, Supplementary Table S7).

MRP-2 inhibition by MK571 was achieved in steatotic HepaRG cells, as the mean (SD) intracellular concentration of sulforhodamine 101 was 152.9% (21.07; *n* = 4 experiments) of untreated steatotic HepaRG controls. In steatotic HepaRG cells, EPL or PPC addition did not significantly impact MRP-2 activity compared with untreated controls (Supplementary Fig. S6B, Supplementary Table S8). Only the highest concentration of PI (1 mg/ml) evaluated in steatotic HepaRG cells significantly increased MRP-2 activity versus untreated controls (LS mean difference [95% CI] –12.4 [–20.9 to –3.8], *P* < 0.01) (Supplementary Fig. S6B, Supplementary Table S8).

#### BSEP

Accumulation of 7-β-(4-nitrobenzo-2-oxa-1,3-diazol [NBD])-taurocholate in the canaliculi of cultured cells was used to assess the impact of PL addition on the bile salt export; thus, increased concentrations of this substrate in the canaliculi demonstrate increased BSEP activity.

**Fig. 4:**
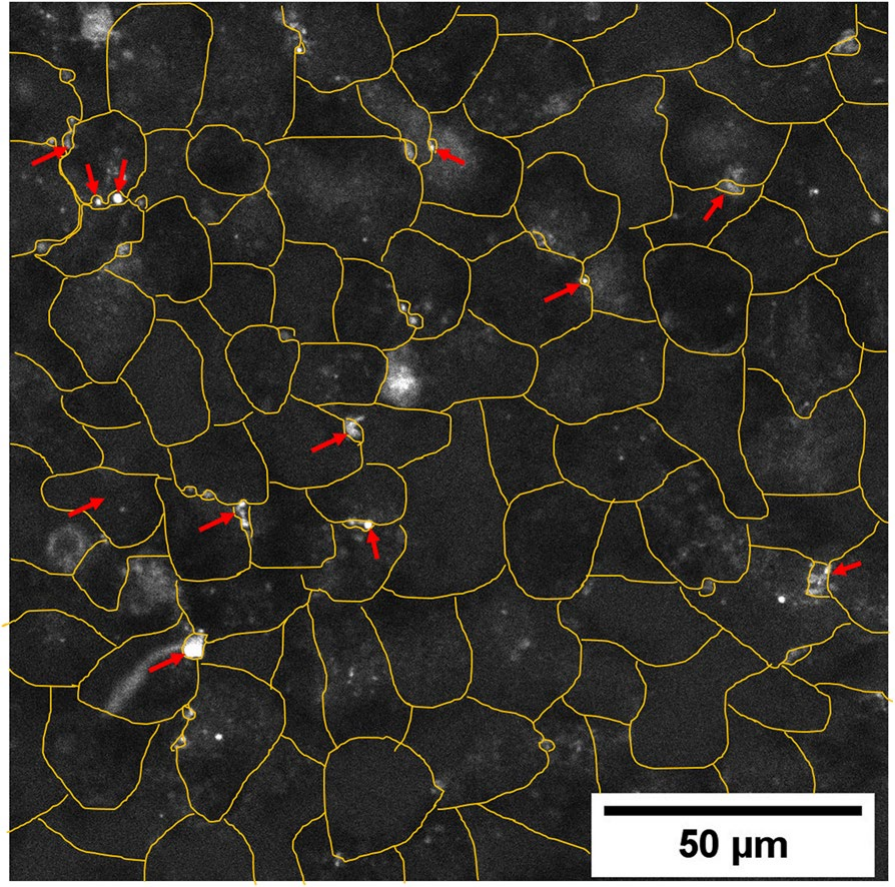
Visualization of BSEP in 0.25 mg/ml EPL treated HepG2 cells. Representative HepG2 cell culture treated with 0.25 mg/ml EPL and visualization of BSEP as accumulation of the substrate 7-beta-NBD-taurocholate in canaliculi. Yellow lines show the individual cells. Red arrows highlight some example spots of canaliculi structures with accumulated fluorescent substrate. BSEP, bile salt export protein; EPL, essential phospholipids

In HepG2 cells, EPL at 0.25 mg/ml significantly increased 7-β-NBD-taurocholate in the canaliculi compared with untreated controls (LS mean difference [95% CI] 96.8 [42.3–151.3], *P* < 0.001) (Fig. 3C, Supplementary Table S6). The addition of PPC to HepG2 cells resulted in a marked increase in bile salt export versus controls at both concentrations evaluated (LS mean difference [95% CI]: 0.1 mg/ml 107.7 [53.3–162.2], *P* < 0.001; 1 mg/ml 168.4 [114.0–222.9], *P* < 0.001) (Fig. 3C, Supplementary Table S6). Figure 4 depicts the accumulation of BSEP in the canaliculi of HepG2 cells exposed to 0.25 mg/ml EPL versus controls. PI addition to HepG2 cells had no significant effect on bile salt export (Fig. 3C, Supplementary Table S6).

There was no significant effect of EPL, PPC, or PI at the concentrations evaluated in HepaRG cell cultures on BSEP activity versus untreated controls (Supplementary Fig. S5C, Supplementary Table S7). In steatotic HepaRG cells, EPL at 0.25 mg/ml significantly increased 7-beta-NBD-taurocholate in the canaliculi versus controls (LS mean difference [95% CI] 116.5 [21.0–212.0], *P* < 0.05) (Supplementary Fig. S6C, Supplementary Table S8).

**Fig. 5:**
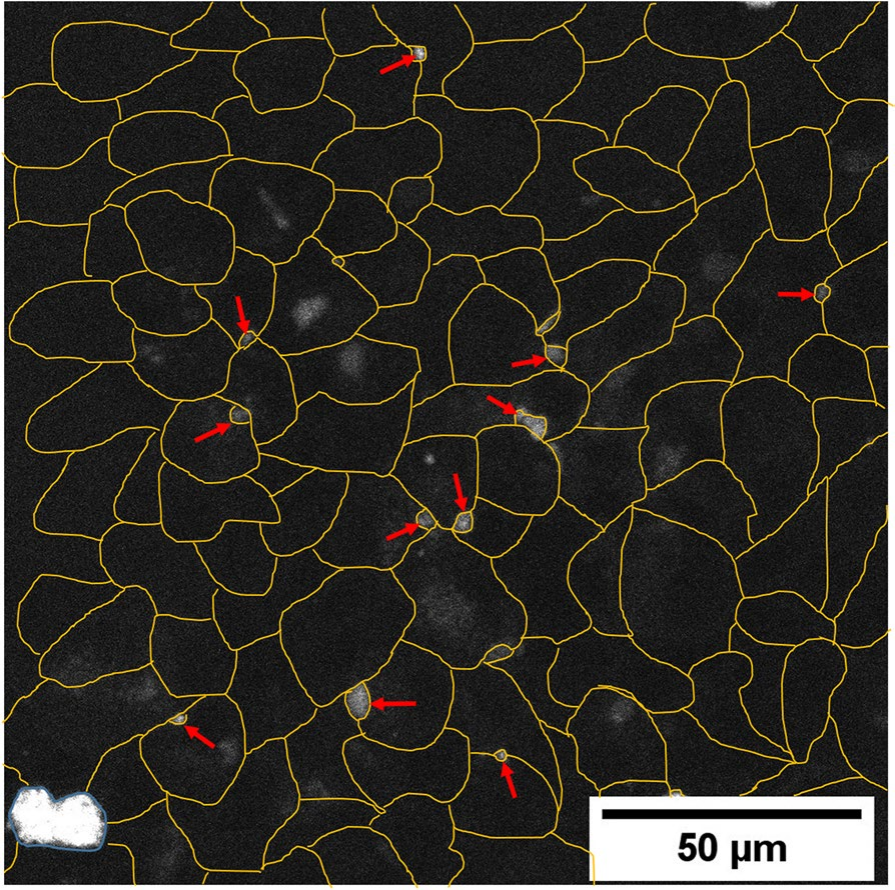
Visualization of BSEP in 0.25 mg/ml EPL treated steatotic HepaRG cells. Representative steatotic HepaRG cell cultures treated with 0.25 mg/ml EPL and visualization of BSEP as accumulation of the substrate 7-beta-NBD-taurocholate in canaliculi. Yellow lines show the individual cells. Red arrows highlight some example spots of canaliculi structures with accumulated fluorescent substrate. The blue circled area with high fluorescence intensity is a dead cell where the fluorescence substrate accumulates. BSEP, bile salt export protein; EPL, essential phospholipids

The addition of PPC to steatotic HepaRG cells increased bile salt export versus controls at both concentrations evaluated (LS mean difference [95% CI]: 0.1 mg/ml 120.3 [24.8–215.8], *P* < 0.05; 1 mg/ml 183.5 [87.9–279.0], *P* < 0.001; Supplementary Fig. S6C, Supplementary Table S8). Figure 5 depicts accumulation of BSEP in the canaliculi of steatotic HepaRG cells exposed to 0.25 mg/ml EPL. PI addition to steatotic HepaRG cells had no significant effect on bile salt export (Supplementary Fig. S6C, Supplementary Table S8).

#### P-GP

The model substrate used for evaluating P-GP transport activity was calcein acetoxymethyl (calcein-am). Increased P-GP activity is demonstrated by decreased intracellular concentrations of this substrate. As a positive control, the P-GP inhibitor PSC833 was added to each cell line in the absence of PL.

As a percentage of untreated HepG2 cells, the mean (SD) intracellular concentration of calcein-am was 140.17 (46.05; *n* = 4 experiments) in the presence of PSC833, indicating P-GP inhibition. All 3 PL preparations added to HepG2 cells in vitro significantly increased P-GP-mediated transport at all concentrations evaluated compared with control (Fig. 3D, Supplementary Table S6). With EPL addition, LS mean differences (95% CI) were: 0.1 mg/ml –32.3 (–42.4 to –22.2), *P* < 0.001; 0.25 mg/ml –41.7 (–51.8 to –31.6). Following PPC addition, LS mean differences (95% CI) were: 0.1 mg/ml –20.1 (–30.2 to –10.0), *P* < 0.001; 1.0 mg/ml –47.2 (–57.3 to –37.1). For PI addition, LS mean differences (95% CI) were: 0.1 mg/ml –32.4 (–42.5 to –22.3), *P* < 0.001; 1.0 mg/ml –43.3 (–53.4 to –33.2).

The mean (SD) intracellular concentration of calcein-am was 207.92% (81.45; *n* = 4 experiments) of untreated HepaRG controls, in the presence of PSC833, indicating P-GP inhibition. Addition of either EPL or PPC at the concentrations evaluated had no significant effects on P-GP transport activity in HepaRG cells versus controls (Supplementary Fig. S5D, Supplementary Table S7). PI addition to HepaRG cells significantly increased P-GP activity only at the 1 mg/ml concentration (LS mean difference [95% CI] –23.2 [–36.1 to –10.4], *P* < 0.001) compared with controls (Supplementary Fig. S5D, Supplementary Table S7).

P-GP inhibition by PSC833 was achieved in steatotic HepaRG cells, as the mean (SD) intracellular concentration of calcein-am was 262.1% (9.86; *n* = 4 experiments) of untreated steatotic HepaRG controls. In steatotic HepaRG cells, EPL and PPC at the concentrations evaluated had no significant effects on P-GP transport activity versus controls (Supplementary Fig. S6D, Supplementary Table S8). PI addition to steatotic HepaRG cells significantly increased P-GP activity only at the 1 mg/ml concentration (LS mean difference [95% CI] –32.6 [–57.0 to –8.1], *P* < 0.01) compared with controls (Supplementary Fig. S6D, Supplementary Table S8).

## Discussion

These investigations of the mechanism of action of EPL and 2 component PLs in human hepatocyte cell lines demonstrated increased membrane fluidity, a significant reduction in apoptosis, and increased hepatocellular extracellular transport involving certain transport proteins, including BSEP. Most of these effects were demonstrated in the HepG2 cell line, whereas minimal effects were observed in the HepaRG and steatotic HepaRG cell lines. This work represents a significant advance in the understanding of the mode of action of EPL as it is the first investigation of EPL (Essentiale®) across different human hepatocyte cell lines.

More than 90% of orally administered EPL is absorbed within 24 h in animals and humans [12, 48, 49]. In rats, most of the EPL dose is hydrolysed during absorption, following which ~ 50% is reacylated to the original model [49, 50]. A wide range of in-vitro, preclinical and clinical investigations demonstrate the hepatoprotective effects of EPL [10, 12–14]. However, a significant data gap exists on the mechanism of action of EPL in hepatocytes. Thus, the present in-vitro studies explored certain pathways as potential targets for EPL, PPC and PI, utilizing 3 human hepatocyte cell lines.

Using anisotropy as an exploratory approach to evaluate membrane fluidity, EPL, PPC, and PI produced a marked increase in membrane fluidity in HepG2 cells versus untreated controls. No differences in membrane fluidity were seen with any PL preparation in HepaRG cells. Only PI at the highest concentration tested increased membrane fluidity in steatotic HepaRG cells. The increase in membrane fluidity appeared to be greater with PI versus EPL and PPC, although the data were variable, indicating that PI has a strong membrane fluidizing effect. Several preclinical studies with EPL reported restoration and strengthening of membrane structure and increased membrane fluidity [12]. PLs are critical components of all cell membranes [20, 51]. PC and PE are major components of plasma membranes and are involved in many cellular processes. A change in the PC: PE ratio occurs in various liver diseases such as NAFLD, liver failure and impaired regeneration [52], and a high PC:PE ratio is a negative predictor of disease progression. Interestingly, membrane fluidity was lower in liver preparations from patients with liver damage, including fatty liver, chronic active hepatitis or cirrhosis versus those from healthy controls [53]. Moreover, the severity of liver disease positively correlated with membrane fluidity [53]. Thus, increasing membrane fluidity of hepatocytes by EPL or 2 of its component PLs, may potentially improve hepatocyte function, and provides further evidence for the mechanism of EPL in improving liver health in humans [13].

Histopathology [54] and biochemical assessments clearly indicate a role for apoptosis in NAFLD and its progression [55]. In the current analyses, EPL, PPC or PI addition to human hepatocyte cell lines did not induce apoptosis. The hepatotoxicant tamoxifen induces apoptosis in hepatocytes [43, 44]. Thus, the 3 cell lines in the present evaluations responded to tamoxifen as a concentration-dependent increase in apoptosis was observed, as expected. EPL, PPC, and PI addition to HepG2 cells significantly decreased tamoxifen-induced apoptosis versus untreated controls. In tamoxifen-treated HepaRG cells or steatotic HepRG cells, addition of any of the 3 PLs did not impact apoptosis under the experimental conditions. These in-vitro data in HepG2 cells support the findings of several other in-vitro studies and preclinical studies evaluating the protective effects of PLs in liver damage [12]. For example, in rats with alcohol-induced liver damage, PPC and dilinoleoylphosphatidylcholine (main component of PPC) decreased alcohol-induced increases in hepatic apoptosis and caspase-3 activity; the latter correlated with the percentage of apoptotic hepatocytes [21]. An in-vitro study in the HepG2 cell line demonstrated that dilinoleoylphosphatidylcholine decreased ethanol-induced apoptosis [56]. Importantly, the presence of a by-product of caspase in serum strongly correlated with NASH [57]. The hepatoprotective effects of these PLs provide further support for apoptosis as a therapeutic target in NAFLD treatment. Indeed, the pan-caspase inhibitor, emricasan, markedly reduced hepatic apoptosis and decreased liver injury and inflammation in mice with NASH [58].

Another critical aspect of the liver is hepatocyte membrane transport function. Bile salts, cholesterol and PC are transported across the apical canalicular membrane of hepatocyte by ATP-binding cassette transporters. All of the transporters evaluated in the present investigations are members of the ATP-binding cassette transporter family [24]. Transporters of bile acids are potentially involved in a wide range of liver disorders, including NAFLD [24–26] and NASH [59]. In the present evaluations, PL addition to HepG2 cells in culture did significantly increase transporter-mediated function, i.e., P-GP (EPL, PPC, and PI), BCRP (EPL and PI), BSEP (EPL and PPC); although the MRP-2 transporter was not affected by any PL preparation. In contrast, in HepaRG cells, MRP-2-mediated transport was significantly increased by all 3 PL preparations, and P-GP activity was increased by PI addition. In steatotic HepaRG cells, significant increases in transporter activity were observed in response to PI (MRP-2 and PG-P) and with EPL (BSEP) or PPC (BSEP). Several preclinical studies also suggested that the excretory capacity of the liver is improved by EPL [12]. The canalicular transporter, MRP-2, was downregulated in obese Zucker rats with defective leptin signaling possibly resulting in accumulation of toxic metabolites [26]. BSEP as a target for NAFLD treatment is of particular interest, as this transporter is the rate limiting step for bile acid efflux; thus, impacting this transporter may have a role in steatohepatitis. Indeed, mice overexpressing BSEP have increased biliary lipid secretion and are protected from steatosis when fed an atherogenic diet [60]. Moreover, BSEP over-expression lowered hepatic lipid accumulation, but not inflammation, in mice fed with a methionine–choline deficient diet (a model for steatohepatitis) [61]. However, there are other bile acid transport proteins (i.e. ATP-binding cassette subfamily C member, organic solute transporter β, solute carrier family 10 member 1, and solute carrier organic transporter family members 1a1 and 1b1), which are disrupted in a mouse model of NASH [59]. Intrahepatic expression of BSEP was downregulated during NAFLD progression, suggesting that BSEP might be involved in the pathogenesis of NAFLD/NASH [62]. Some studies have reported a positive association between BSEP variants and increased serum triglycerides and cholesterol levels and obesity in humans [63].

Clear differences in the results between the different cell lines used in these investigations were seen. One reason for such differences might be that differentiated HepaRG cells exhibit different levels of expression of drug-metabolizing enzymes and drug transporters compared with the HepG2 cell line [32]. Furthermore, whole genome gene expression profiles of HepaRG cells and HepG2 cells indicated that HepaRG cells globally transcribed genes which were more similar to primary human hepatocytes and human liver tissue samples than to HepG2 cells [33]. As each cell line has its own specific characteristics, the data from all cell lines should be considered to obtain an overview of the effects of PLs on liver function in vitro.

NAFLD and its progression is very complex and involves numerous biochemical pathways, and only 3 potential targets have been explored in the current in-vitro studies. Two other pathways disrupted in NAFLD, which are also of potential interest as targets for therapy, are inflammation [64] and lipid metabolism [65]. There is evidence from clinical and preclinical studies that EPL administration impacts these 2 processes in vivo [12, 66]. Further work investigating the impact of EPL, PPC and PI on pro-inflammatory cytokines, and on lipid-metabolizing enzymes in vitro will be reported separately.

These investigations have several strengths and weaknesses. The major strength is that several human hepatocyte cell lines were used, which are directly applicable for extrapolation to human studies. Moreover, such in-vitro techniques are ideally suited for mechanistic work of compounds of interest. In contrast, all cell lines used were immortal cancer cell lines, which are unlikely to fully represent normal liver function. Although maximum, noncytotoxic concentrations of each PL investigated were evaluated, how these concentrations in vitro relate to in-vivo concentrations, particularly at the level of the hepatocyte are unknown.

## Conclusions

In summary, in-vitro investigations in HepG2, HepaRG, steatotic HepaRG to evaluate the effects of EPL, PPC and PI provided valuable insights into the mechanism of action of EPL, which has hepatoprotective effects in patients with NAFLD and other liver conditions. The in-vitro results demonstrated increased membrane fluidity, decreased apoptosis, and increased function of hepatocellular extracellular transporters involved in bile secretion, all of which may potentially improve liver function. This first investigation of EPL (Essentiale®) in human hepatocyte cell lines is an important contribution to understanding the mechanism of action of EPL. Further in-vitro work on the effects of EPL on pro-inflammatory cytokines and lipid-metabolizing enzymes will also provide further insights on the hepatoprotective properties of these preparations.

## Supplementary Information

**Additional file 1:** **Supplementary Table S1.** Cytotoxicity of EPL, PPC and PI in HepaRG cell line. **Supplementary Table S2.** Statistical analyses of the effects of EPL, PPC and PI on anisotropy in the HepG2, HepaRG and steatotic HepaRG cell lines. **Supplementary Table S3.** Statistical analyses of the effects of EPL, PPC and PI on apoptosis in the HepG2 cell line. **Supplementary Table S4.** Statistical analyses of the effects of EPL, PPC and PI on apoptosis in the HepaRG cell line. **Supplementary Table S5.** Statistical analyses of the effects of EPL, PPC and PI on apoptosis in the steatotic HepaRG cell line. **Supplementary Table S6.** Statistical analyses of the effects of EPL, PPC and PI on hepatocellular transport protein activity in the HepG2 cell line. **Supplementary Table S7.** Statistical analyses of the effects of EPL, PPC and PI on hepatocellular transport protein activity in the HepaRG cell line. **Supplementary Table S8.** Statistical analyses of the effects of EPL, PPC and PI on hepatocellular transport protein activity in the steatotic HepaRG cell line. **Supplementary Fig. S1.** Gating strategy to evaluate caspase-3/-7 activity in apoptotic cells by flow cytometry. **Supplementary Fig. S2.** Effect of EPL, PPC and PI on anisotropy in the HepaRG and steatotic HepaRG cell lines. **Supplementary Fig. S3.** Effect of EPL, PPC and PI on apoptosis in the HepaRG cell line. **Supplementary Fig. S4.** Effect of EPL, PPC and PI on apoptosis in the steatotic HepaRG cell line. **Supplementary Fig. S5.** Effect of EPL, PPC and PI on hepatocellular transport protein activity in the HepaRG cell line. **Supplementary Fig S6.** Effect of EPL, PPC and PI on hepatocellular transport protein activity in the steatotic HepaRG cell line.

## Data Availability

Qualified researchers may request access to the data and related study documents including the study report, protocol with any amendments, statistical analysis plan, and dataset specifications. Further details on Sanofi's data sharing criteria, eligible studies, and process for requesting access can be found at: https://www.clinicalstudydatarequest.com/.

## Abbreviations

BCRP: Breast cancer resistance protein
BSEP: Bile salt export protein; calcein-am, calcein acetoxymethyl
CI: Confidence interval
DMSO: Dimethyl sulfoxide
DPH: 1,6-Diphenyl-1,3,5-hexatriene
EPL: Essential phospholipids
FACS: Fluorescence-activated cell sorting
FBS: Fetal bovine serum
LS: Least-square
MRP-2: Multidrug resistance-associated protein 2
NAFLD: Nonalcoholic fatty liver disease
NASH: Nonalcoholic steatohepatitis
NBD: 4-Nitrobenzo-2-oxa-1,3-diazol
PBS: Phosphate buffered saline
PE: Phosphatidylethanolamine
P-GP: P-glycoprotein
PI: Phosphatidylinositol
PLs: Phospholipids
PPC: Polyenylphosphatidylcholine
SD: Standard deviation
