# Supplementary material for: Essential phospholipids decrease apoptosis and increase membrane transport in human hepatocyte cell lines
Source: Lipids Health Dis. 2022 Sep 24;21:91. doi: 10.1186/s12944-022-01698-8 (PMC9508738; doi:10.1186/s12944-022-01698-8)
Supplement: Supplementary file 1 — Additional file 1: Supplementary Table S1. Cytotoxicity of EPL, PPC and PI in HepaRG cell line. Supplementary Table S2. Statistical analyses of the effects of EPL, PPC and PI on anisotropy in the HepG2, HepaRG and steatotic HepaRG cell lines. Supplementary Table S3. Statistical analyses of the effects of EPL, PPC and PI on apoptosis in the HepG2 cell line. Supplementary Table S4. Statistical analyses of the effects of EPL, PPC and PI on apoptosis in the HepaRG cell line. Supplementary Table S5. Statistical analyses of the effects of EPL, PPC and PI on apoptosis in the steatotic HepaRG cell line. Supplementary Table S6. Statistical analyses of the effects of EPL, PPC and PI on hepatocellular transport protein activity in the HepG2 cell line. Supplementary Table S7. Statistical analyses of the effects of EPL, PPC and PI on hepatocellular transport protein activity in the HepaRG cell line. Supplementary Table S8. Statistical analyses of the effects of EPL, PPC and PI on hepatocellular transport protein activity in the steatotic HepaRG cell line. Supplementary Fig. S1. Gating strategy to evaluate caspase-3/-7 activity in apoptotic cells by flow cytometry. Supplementary Fig. S2. Effect of EPL, PPC and PI on anisotropy in the HepaRG and steatotic HepaRG cell lines. Supplementary Fig. S3. Effect of EPL, PPC and PI on apoptosis in the HepaRG cell line. Supplementary Fig. S4. Effect of EPL, PPC and PI on apoptosis in the steatotic HepaRG cell line. Supplementary Fig. S5. Effect of EPL, PPC and PI on hepatocellular transport protein activity in the HepaRG cell line. Supplementary Fig S6. Effect of EPL, PPC and PI on hepatocellular transport protein activity in the steatotic HepaRG cell line. [file 12944_2022_1698_MOESM1_ESM.docx]

**`Supplementary Table S1** Cytotoxicity of EPL, PPC and PI in HepaRG cell line

| Cell viability  (% of untreated cells) | EPL concentration mg/ml | | | | | | | |
| --- | --- | --- | --- | --- | --- | --- | --- | --- |
|  | **0.01** | **0.02** | **0.1** | **0.2** | **1** | **2** | **10** | **20** |
| Mean  (SD) [range] | 106.33  (0.85)  [105.2–107.2] | 101.00 (2.23)  [98.0–103.3] | 105.62  (1.76)  [103.4–107.5] | 107.73  (1.43)  [105.7–109.1] | 116.94  (0.98)  [115.8–118.1] | 118.56  (2.43)  [115.5–120.8] | 122.33  (1.07)  [121.3–123.6] | 123.04  (0.75)  [122.2–124.0] |
|  | **PPC concentration mg/ml** | | | | | | | |
|  | **0.01** | **0.02** | **0.1** | **0.2** | **1** | **2** | **10** | **20** |
| Mean  (SD) [range] | 98.84  (2.88)  [94.9–101.1] | 95.94  (1.05)  [94.9–97.4] | 101.51  (1.21)  [100.0–102.7] | 101.42  (0.82)  [100.7–102.4] | 106.23  (0.50)  [105.6–106.8] | 108.04  (0.72)  [107.1–108.7] | 122.32  (4.23)  [119.7–128.6] | 129.10  (6.04)  [124.8–138.0] |
|  | **PI concentration mg/ml** | | | | | | | |
|  | **0.01** | **0.02** | **0.1** | **0.2** | **1** | **2** | **10** | **20** |
| Mean  (SD) [range] | 101.80  (1.07)  [100.4–102.7] | 98.90  (1.28)  [97.0–99.9] | 98.75  (1.23)  [97.6–100.5] | 102.64  (1.30)  [101.4–104.3] | 112.33  (2.05)  [110.9–115.4] | 115.82  (2.15)  [114.3–119.0] | 119.12  (6.60)  [114.2–128.6] | 123.06  (5.43)  [118.4–130.9] |

Values shown are mean ± SD (range) as percentage of untreated cells (no PL addition) for 4 separate experiments; n = 3 replicate wells for each concentration of each compound per experiment

EPL: essential phospholipids; PI: phosphatidylinositol; PL: phospholipid; PPC: polyenylphosphatidylcholine; SD: standard deviation

**Supplementary Table S2** Statistical analyses of the effects of EPL, PPC and PI on anisotropy in the HepG2, HepaRG and steatotic HepaRG cell lines.

| Cell line | Statistic | Untreated cells  (n = 4) | 0.1 mg/ml EPL  (n = 3) | 0.25 mg/ml  EPL  (n = 3) | 0.1 mg/ml PPC  (n = 3) | 1 mg/ml  PPC  (n = 3) | 0.1 mg/ml  PI  (n = 3) | 0.1 mg/ml  PI  (n = 3) | Overall treatment effect *P*-value |
| --- | --- | --- | --- | --- | --- | --- | --- | --- | --- |
| HepG2 | LS mean (SE) | 0.05975  (0.00239) | 0.02208  (0.00276) | 0.00209  (0.00276) | 0.02983  (0.00276) | 0.01150  (0.00276) | 0.01888  (0.00239) | –0.00801  (0.00276) | < 0.0001 |
|  | LS mean difference (SE) |  | –0.03768  (0.00366) | –0.05767  (0.00366) | –0.02992  (0.00366) | –0.04826  (0.00366) | –0.04087  (0.00366) | –0.06776  (0.00366) |  |
|  | *P*-value |  | < 0.0001 | < 0.0001 | < 0.0001 | <0.0001 | < 0.0001 | < 0.0001 |  |
| HepaRG* | LS mean (SE) | 0.05839  (0.00608) | 0.04803  (0.00608) | 0.04224  (0.00608) | 0.05091  (0.00608) | 0.03988  (0.00608) | 0.04513  (0.00608) | 0.02687  (0.00608) | 0.0463 |
|  | LS mean difference (SE) |  | –0.01035  (0.00860) | –0.01614  (0.00860) | –0.00747  (0.00860) | –0.01851  (0.00860) | –0.01326  (0.00860) | –0.03151  (0.00860) |  |
|  | *P*-value |  | 0.6875 | 0.2792 | 0.8891 | 0.1744 | 0.4609 | 0.0073 |  |
| HepaRG steotatic* | LS means (SE) | 0.00553  (0.00221) | 0.00309  (0.00221) | –0.00000  (0.00221) | 0.00515  (0.00221) | 0.00345  (0.00221) | 0.00010  (0.00221) | –0.00360  (0.00221) | 0.0823 |
|  | LS mean difference (SE) |  | –0.00244  (0.00312) | –0.00554  (0.00312) | –0.00038  (0.00312) | –0.00208  (0.00312) | –0.00543  (0.00312) | –0.00914  (0.00312) |  |
|  | *P*-value |  | 0.9262 | 0.3287 | 1.0000 | 0.9628 | 0.3464 | 0.0374 |  |

LS means were estimated using an ANOVA including treatment groups as fixed factors. Pairwise comparison has been estimated using a Dunnett’s adjustment.

*n = 4 for all groups. This table complements Fig.1 and Supplementary Fig. S2.

EPL, essential phospholipids; LS, least-square; PI, phosphatidylinositol; PPC, polyenylphosphatidylcholine; SE, standard error

**Supplementary Table S3** Statistical analyses of the effects of EPL, PPC and PI on apoptosis in the HepG2 cell line.

| Parameter | Statistic | Tamoxifen concentration | Untreated cells  (n = 4) | 0.1 mg/ml EPL  (n = 4) | 0.25 mg/ml  EPL  (n = 4) | | 0.1 mg/ml  PPC  (n = 4) | | 1 mg/ml  PPC  (n = 4) | | 0.1 mg/ml  PI  (n = 4) | | 0.1 mg/ml  PI  (n = 4) | | Overall treatment effect *P*-value | |
| --- | --- | --- | --- | --- | --- | --- | --- | --- | --- | --- | --- | --- | --- | --- | --- | --- |
| Sytox- negative cells (%) | LS mean (SE) | 0 µM | 100.0 (4.7) | 93.1 (4.7) | 89.8 (4.7) | | 102.0 (4.7) | | 88.8 (4.7) | | 93.5 (4.7) | | 93.8 (4.7) | | < 0.0001^a^  < 0.0001^b^  0.0115^c^ | |
|  | LS mean difference (SE) |  |  | –6.9 (6.6) | –10.2 (6.6) | | 2.0 (6.6) | | –11.2 (6.6) | | –6.5 (6.6) | | –6.2 (6.6) | |  |  |
|  | *P*-value |  |  | 1.0000 | 0.9918 | | 1.0000 | | 0.9781 | | 1.0000 | | 1.0000 | |  |  |
|  | LS means (SE) | 42 µM | 100.5 (4.7) | 83.6 (4.7) | 82.3 (4.7) | | 96.1 (4.7) | | 81.6 (4.7) | | 90.1 (4.7) | | 76.8 (4.7) | |  |  |
|  | LS mean difference (SE) |  |  | –17.0 (6.6) | –18.3 (6.6) | | –4.4 (6.6) | | –19.0 (6.6) | | –10.5 (6.6) | | –23.7 (6.6) | |  |  |
|  | *P*-value |  |  | 0.5617 | 0.4234 | | 1.0000 | | 0.3571 | | 0.9898 | | 0.0729 | |  |  |
|  | LS mean (SE) | 55 µM | 129.2 (4.7) | 90.5 (4.7) | 86.1 (4.7) | | 116.8 (4.7) | | 89.9 (4.7) | | 102.8 (4.7) | | 80.1 (4.7) | |  |  |
|  | LS mean difference (SE) |  |  | –38.8 (6.6) | –43.2 (6.6) | | –12.5 (6.6) | | –39.3 (6.6) | | –26.5 (6.6) | | –49.1 (6.6) | |  |  |
|  | *P*-value |  |  | < 0.0001 | < 0.0001 | | 0.9414 | | < 0.0001 | | 0.0227 | | < 0.0001 | |  |  |
| Sytox-positive cells (dead cells) (%) | LS mean (SE) | 0 µM | 100.0 (56.7) | 106.5 (56.7) | 125.2 (56.7) | | 125.4 (56.7) | | 138.6 (56.7) | | 118.0 (56.7) | | 147.7 (56.7) | | < 0.0001^a^  < 0.0001^b^  < 0.0001^c^ | |
|  | LS mean difference (SE) |  |  | 6.5 (80.3) | 25.2 (80.3) | | 25.3 (80.3) | | 38.6 (80.3) | | 18.0 (80.3) | | 47.7 (80.3) | |  |  |
|  | *P*-value |  |  | 1.0000 | 1.0000 | | 1.0000 | | 1.0000 | | 1.0000 | | 1.0000 | |  |  |
|  | LS mean (SE) | 42 µM | 742.8 (56.7) | 560.5 (56.7) | 702.8 (56.7) | | 710.1 (56.7) | | 728.9 (56.7) | | 725.0 (56.7) | | 378.9 (56.7) | |  |  |
|  | LS mean difference (SE) |  |  | –182.4 (80.3) | –40.0 (80.3) | | –32.7 (80.3) | | –14.0 (80.3) | | –17.8 (80.3) | | –364.0 (80.3) | |  |  |
|  | *P*-value |  |  | 0.7654 | 1.0000 | | 1.0000 | | 1.0000 | | 1.0000 | | 0.0042 | |  |  |
|  | LS mean (SE) | 55 µM | 1576.9 (56.7) | 1124.1 (56.7) | 1283.2 (56.7) | | 1204.5 (56.7) | | 1160.8 (56.7) | | 1188.8 (56.7) | | 531.6 (56.7) | |  |  |
|  | LS mean difference (SE) |  |  | –452.8 (80.3) | –293.7 (80.3) | | –372.4 (80.3) | | –416.1 (80.3) | | –388.1 (80.3) | | –1045.3 (80.3) | |  |  |
|  | *P*-value |  |  | < 0.0001 | 0.0599 | 0.0029 | | 0.0004 | | 0.0015 | | < 0.0001 | |  | |  |

LS means were estimated using an ANOVA including treatment group and tamoxifen level as fixed factors and treatment group and tamoxifen interaction. Pairwise comparison has been estimated using a Turkey’s adjustment

^a^overall treatment effect, ^b^overall tamoxifen levels effect, ^c^overall treatment + tamoxifen levels interaction effect. This table complements Fig. 2.

EPL, essential phospholipids; LS, least-square; PI, phosphatidylinositol; PPC, polyenylphosphatidylcholine; SE, standard error

**Supplementary Table S4** Statistical analyses of the effects of EPL, PPC and PI on apoptosis in the HepaRG cell line.

| Parameter | Statistic | Tamoxifen concentration | Untreated cells  (n = 4) | 0.1 mg/ml EPL  (n = 4) | 0.25 mg/ml  EPL  (n = 4) | 0.1 mg/ml  PPC  (n = 4) | 1 mg/ml  PPC  (n = 4) | 0.1 mg/ml  PI  (n = 4) | 0.1 mg/ml  PI  (n = 4) | Overall treatment effect *P*-value |
| --- | --- | --- | --- | --- | --- | --- | --- | --- | --- | --- |
| Sytox- negative cells (%) | LS mean (SE) | 0 µM | 100.0 (5.7) | 96.0 (5.7) | 92.5 (5.7) | 102.4 (5.7) | 95.3 (5.7) | 96.7 (5.7) | 110.6 (5.7) | < 0.0001^a^  < 0.0001^b^  0.8203^c^ |
|  | LS mean difference (SE) |  |  | –4.0 (8.1) | –7.6 (8.1) | 2.3 (8.1) | –4.8 (8.1) | –3.4 (8.1) | 10.5 (8.1) |  |
|  | *P*-value |  |  | 1.0000 | 1.0000 | 1.0000 | 1.0000 | 1.0000 | 0.9991 |  |
|  | LS mean (SE) | 45 µM | 136.9 (5.7) | 134.7 (5.7) | 132.7 (5.7) | 155.5 (5.7) | 136.6 (5.7) | 143.3 (5.7) | 151.6 (5.7) |  |
|  | LS mean difference (SE) |  |  | –2.2 (8.1) | –4.2 (8.1) | 18.6 (8.1) | –0.3 (8.1) | 6.4 (8.1) | 14.7 (8.1) |  |
|  | *P*-value |  |  | 1.0000 | 1.0000 | 0.7506 | 1.0000 | 1.0000 | 0.9581 |  |
|  | LS mean (SE) | 60 µM | 142.1 (5.7) | 141.3 (5.7) | 131.2 (5.7) | 164.4 (5.7) | 139.0 (5.7) | 152.2 (5.7) | 151.7 (5.7) |  |
|  | LS mean difference (SE) |  |  | –0.8 (8.1) | –11.0 (8.1) | 22.3 (8.1) | –3.1 (8.1) | 10.1 (8.1) | 9.6 (8.1) |  |
|  | *P*-value |  |  | 1.0000 | 0.9985 | 0.4343 | 1.0000 | 0.9995 | 0.9998 |  |
| Sytox-positive cells (dead cells) (%) | LS mean (SE) | 0 µM | 100.0 (15.8) | 117.3 (15.8) | 140.5 (15.8) | 160.0 (15.8) | 142.7 (15.8) | 170.8 (15.8) | 185.7 (15.8) | < 0.0001^a^  < 0.0001^b^  0.2838^c^ |
|  | LS mean difference (SE) |  |  | 17.3 (22.3) | 40.5 (22.3) | 60.0 (22.3) | 42.7 (22.3) | 70.8 (22.3) | 85.7 (22.3) |  |
|  | *P*-value |  |  | 1.0000 | 0.9572 | 0.4729 | 0.9326 | 0.1955 | 0.0360 |  |
|  | LS means (SE) | 45 µM | 198.6 (15.8) | 255.5 (15.8) | 250.1 (15.8) | 315.4 (15.8) | 260.0 (15.8) | 288.6 (15.8) | 277.8 (15.8) |  |
|  | LS mean difference (SE) |  |  | 56.9 (22.3) | 51.5 (22.3) | 116.9 (22.3) | 61.4 (22.3) | 90.0 (22.3) | 79.2(22.3) |  |
|  | *P*-value |  |  | 0.5721 | 0.7398 | 0.0004 | 0.4289 | 0.0203 | 0.0790 |  |
|  | LS mean (SE) | 60 µM | 263.7 (15.8) | 258.0 (15.8) | 319.5 (15.8) | 363.8 (15.8) | 282.7(15.8) | 346.0 (15.8) | 310.9 (15.8) |  |
|  | LS mean difference (SE) |  |  | –5.7 (22.3) | 55.8 (22.3) | 100.1 (22.3) | 19.0 (22.3) | 82.3 (22.3) | 47.2 (22.3) |  |
|  | *P*-value |  |  | 1.0000 | 0.6076 | 0.0049 | 1.0000 | 0.0551 | 0.8525 |  |

LS means were estimated using an ANOVA including treatment group and tamoxifen level as fixed factors and treatment group and tamoxifen interaction. Pairwise comparison has been estimated using a Turkey’s adjustment

^a^overall treatment effect, ^b^overall tamoxifen levels effect, ^c^overall treatment + tamoxifen levels interaction effect. This table complements Supplementary Fig. S3.

EPL, essential phospholipids; LS, least-square; PI, phosphatidylinositol; PPC, polyenylphosphatidylcholine; SE, standard error

**Supplementary Table S5** Statistical analyses of the effects of EPL, PPC and PI on apoptosis in the steatotic HepaRG cell line.

| Parameter | Statistic | Tamoxifen concentration | Untreated cells  (n = 4) | 0.1 mg/ml EPL  (n = 4) | 0.25 mg/ml  EPL  (n = 4) | 0.1 mg/ml  PPC  (n = 4) | 1 mg/ml  PPC  (n = 4) | 0.1 mg/ml  PI  (n = 4) | 0.1 mg/ml  PI  (n = 4) | Overall treatment effect *P*-value |
| --- | --- | --- | --- | --- | --- | --- | --- | --- | --- | --- |
| Sytox- negative cells (%) | LS mean (SE) | 0 µM | 100.0 (4.3) | 107.8 (4.3) | 105.0 (4.3) | 103.3 (4.3) | 100.3 (4.3) | 100.8(4.3) | 103.5 (4.3) | 0.1045^a^  < 0.0001^b^  0.9097^c^ |
|  | LS mean difference (SE) |  |  | 7.7 (6.1) | 5.0 (6.1) | 3.2 (6.1) | 0.2 (6.1) | 0.7 (6.1) | 3.5 (6.1) |  |
|  | *P*-value |  |  | 0.9994 | 1.0000 | 1.0000 | 1.0000 | 1.0000 | 1.0000 |  |
|  | LS mean (SE) | 45 µM | 118.3 (4.3) | 115.0 (4.3) | 120.8 (4.3) | 109.0 (4.3) | 112.0 (4.3) | 112.3 (4.3) | 111.0 (4.3) |  |
|  | LS mean difference (SE) |  |  | –3.3 (6.1) | 2.5 (6.1) | –9.3 (6.1) | –6.3 (6.1) | –6.0 (6.1) | –7.3 (6.1) |  |
|  | *P*-value |  |  | 1.0000 | 1.0000 | 0.9942 | 1.0000 | 1.0000 | 0.9998 |  |
|  | LS mean (SE) | 60 µM | 122.8 (4.3) | 119.8 (4.3) | 122.3 (4.3) | 112.3 (4.3) | 114.3 (4.3) | 112.5 (4.3) | 109.8 (4.3) |  |
|  | LS mean difference (SE) |  |  | –3.0 (6.1) | –0.5 (6.1) | –10.5 (6.1) | –8.5 (6.1) | –10.3 (6.1) | –13.0 (6.1) |  |
|  | *P*-value |  |  | 1.0000 | 1.0000 | 0.9766 | 0.9979 | 0.9818 | 0.8523 |  |
| Sytox-positive cells (dead cells) (%) | LS mean (SE) | 0 µM | 100.0 (16.1) | 100.7 (16.1) | 111.7 (16.1) | 123.0 (16.1) | 142.7 (16.1) | 138.0 (16.1) | 139.5 (16.1) | 0.2165^a^  < 0.0001^b^  0.8798^c^ |
|  | LS mean difference (SE) |  |  | 0.8 (22.7) | 11.8 (22.7) | 23.0 (22.7) | 42.8 (22.7) | 38.0 (22.7) | 39.5 (22.7) |  |
|  | *P*-value |  |  | 1.0000 | 1.0000 | 1.0000 | 0.9422 | 0.9814 | 0.9724 |  |
|  | LS mean (SE) | 45 µM | 137.5 (16.1) | 151.3 (16.1) | 175.7 (16.1) | 154.8 (16.1) | 176.0 (16.1) | 181.0 (16.1) | 168.0 (16.1) |  |
|  | LS mean difference (SE) |  |  | 13.8 (22.7) | 38.3 (22.7) | 17.3 (22.7) | 38.5 (22.7) | 43.5 (22.7) | 30.5 (22.7) |  |
|  | *P*-value |  |  | 1.0000 | 0.9801 | 1.0000 | 0.9787 | 0.9328 | 0.9986 |  |
|  | LS mean (SE) | 60 µM | 183.7 (16.1) | 191.5 (16.1) | 184.0 (16.1) | 190.5 (16.1) | 197.5 (16.1) | 187.7 (16.1) | 174.2 (16.1) |  |
|  | LS mean difference (SE) |  |  | 7.8 (22.7) | 0.3 (22.7) | 6.8 (22.7) | 13.8 (22.7) | 4.0 (22.7) | –9.5 (22.7) |  |
|  | *P*-value |  |  | 1.0000 | 1.0000 | 1.0000 | 1.0000 | 1.0000 | 1.0000 |  |

LS means were estimated using an ANOVA including treatment group and tamoxifen level as fixed factors and treatment group and tamoxifen interaction. Pairwise comparison has been estimated using a Turkey’s adjustment

^a^overall treatment effect, ^b^overall tamoxifen levels effect, ^c^overall treatment + tamoxifen levels interaction effect. This table complements Supplementary Fig. S4.

EPL, essential phospholipids; LS, least-square; PI, phosphatidylinositol; PPC, polyenylphosphatidylcholine; SE, standard error

**Supplementary Table S6** Statistical analyses of the effects of EPL, PPC and PI on hepatocellular transport protein activity in the HepG2 cell line.

| Parameter | Statistic | Untreated cells  (n = 5) | 0.1 mg/ml EPL  (n = 5) | 0.25 mg/ml  EPL  (n = 5) | 0.1 mg/ml PPC  (n = 5) | 1 mg/ml  PPC  (n = 5) | 0.1 mg/ml  PI  (n = 5) | 0.1 mg/ml  PI  (n = 5) | Overall treatment effect *P*-value |
| --- | --- | --- | --- | --- | --- | --- | --- | --- | --- |
| Breast cancer resistance protein (%) | LS mean (SE) | 100.0 (2.5) | 83.8 (2.5) | 68.2 (2.5) | 98.6 (2.5) | 96.0 (2.5) | 77.8 (2.5) | 64.0 (2.5) | < 0.0001 |
|  | LS mean difference (SE) |  | –16.2 (3.6) | –31.8 (3.6) | –1.4 (3.6) | –4.0 (3.6) | –22.2 (3.6) | –36.0 (3.6) |  |
|  | *P*-value |  | 0.0006 | < 0.0001 | 0.9975 | 0.7449 | < 0.0001 | < 0.0001 |  |
| Multidrug resistance-associated protein 2 (%)* | LS mean (SE) | 100.0 (4.6) | 89.6 (4.6) | 86.3 (4.6) | 88.8 (4.6) | 83.1 (4.6) | 84.0 (4.6) | 97.9 (4.6) | 0.1011 |
|  | LS mean difference (SE) |  | –10.4 (6.5) | –13.7 (6.5) | –11.3 (6.5) | –16.9 (6.5) | –16.0 (6.5) | –2.1 (6.5) |  |
|  | *P*-value |  | 0.4206 | 0.1883 | 0.3501 | 0.0726 | 0.0968 | 0.9990 |  |
| Bile salt export pump (%)* | LS mean (SE) | 99.8 (13.8) | 150.4 (13.8) | 196.6 (13.8) | 207.6 (13.8) | 268.3 (13.8) | 147.5 (13.8) | 131.5 (13.8) | < 0.0001 |
|  | LS mean difference (SE) |  | 50.6 (19.5) | 96.8 (19.5) | 107.7 (19.5) | 168.4 (19.5) | 47.7 (19.5) | 31.7 (19.5) |  |
|  | *P*-value |  | 0.0755 | 0.0004 | < 0.0001 | < 0.0001 | 0.1010 | 0.4115 |  |
| P-glycoprotein (%)* | LS mean (SE) | 100.0 (2.6) | 67.7 (2.6) | 58.3 (2.6) | 79.9 (2.6) | 52.8 (2.6) | 67.6 (2.6) | 56.7 (2.6) | < 0.0001 |
|  | LS mean difference (SE) |  | –32.3 (3.6) | –41.7 (3.6) | –20.1 (3.6) | –47.2 (3.6) | –32.4 (3.6) | –43.3 (3.6) |  |
|  | *P*-value |  | < 0.0001 | < 0.0001 | < 0.0001 | < 0.0001 | < 0.0001 | < 0.0001 |  |

LS means were estimated using an ANOVA including treatment groups as fixed factors. Pairwise comparison has been estimated using a Dunnett’s adjustment.

*n = 4 for all groups. This table complements Fig. 3.

EPL, essential phospholipids; LS, least-square; PI, phosphatidylinositol; PPC, polyenylphosphatidylcholine; SE, standard error

**Supplementary Table S7** Statistical analyses of the effects of EPL, PPC and PI on hepatocellular transport protein activity in the HepaRG cell line.

| Parameter | Statistic | Untreated cells  (n = 4) | 0.1 mg/ml EPL  (n = 4) | 0.25 mg/ml  EPL  (n = 4) | 0.1 mg/ml PPC  (n = 4) | 1 mg/ml  PPC  (n = 4) | 0.1 mg/ml  PI  (n = 4) | 0.1 mg/ml  PI  (n = 4) | Overall treatment effect *P*-value |
| --- | --- | --- | --- | --- | --- | --- | --- | --- | --- |
| Breast cancer resistance protein (%) | LS mean (SE) | 100.0  (3.2) | 93.4 (3.2) | 91.8 (3.2) | 97.8 (3.2) | 94.2 (3.2) | 97.1 (3.2) | 94.2 (3.2) | 0.5691 |
|  | LS mean difference (SE) |  | –6.7 (4.5) | –8.2 (4.5) | –2.2 (4.5) | –5.8 (4.5) | –2.9 (4.5) | –5.8 (4.5) |  |
|  | *P*-value |  | 0.5036 | 0.3026 | 0.9911 | 0.6269 | 0.9669 | 0.6364 |  |
| Multidrug resistance-associated protein 2 (%) | LS mean (SE) | 100.0 (2.2) | 89.6 (2.2) | 87.1 (2.2) | 94.9 (2.2) | 85.1 (2.2) | 90.4 (2.2) | 77.7 (2.2) | < 0.0001 |
|  | LS mean difference (SE) |  | –10.4 (3.1) | –12.9 (3.1) | –5.1 (3.1) | –14.9 (3.1) | –9.6 (3.1) | –22.3 (3.1) |  |
|  | *P*-value |  | 0.0154 | 0.0025 | 0.4017 | 0.0006 | 0.0277 | < 0.0001 |  |
| Bile salt export pump (%) | LS mean (SE) | 100.0 (15.6) | 122.7 (15.6) | 130.6 (15.6) | 126.6 (15.6) | 142.3 (15.6) | 131.1 (15.6) | 133.0 (15.6) | 0.6407 |
|  | LS mean difference (SE) |  | 22.7 (22.1) | 30.5 (22.1) | 26.5 (22.1) | 42.3 (22.1) | 31.1 (22.1) | 33.0 (22.1) |  |
|  | *P*-value |  | 0.8024 | 0.5633 | 0.6876 | 0.2611 | 0.5461 | 0.4902 |  |
| P-glycoprotein (%) | LS mean (SE) | 100.0 (3.3) | 91.8 (3.3) | 94.5 (3.3) | 110.0 (3.3) | 103.5 (3.3) | 98.8 (3.3) | 76.8 (3.3) | < 0.0001 |
|  | LS mean difference (SE) |  | –8.3 (4.6) | –5.5 (4.6) | 10.0 (4.6) | 3.5 (4.6) | –1.2 (4.6) | –23.2 (4.6) |  |
|  | *P*-value |  | 0.3189 | 0.6928 | 0.1675 | 0.9341 | 0.9998 | 0.0003 |  |

LS means were estimated using an ANOVA including treatment groups as fixed factors. Pairwise comparison has been estimated using a Dunnett’s adjustment.

This table complements Supplementary Fig. S5.

EPL, essential phospholipids; LS, least-square; PI, phosphatidylinositol; PPC, polyenylphosphatidylcholine; SE, standard error

**Supplementary Table S8** Statistical analyses of the effects of EPL, PPC and PI on hepatocellular transport protein activity in the steatotic HepaRG cell line.

| Parameter | Statistic | Untreated cells  (n = 4) | 0.1 mg/ml EPL  (n = 4) | 0.25 mg/ml  EPL  (n = 4) | 0.1 mg/ml PPC  (n = 4) | 1 mg/ml  PPC  (n = 4) | 0.1 mg/ml  PI  (n = 4) | 0.1 mg/ml  PI  (n = 4) | Overall treatment effect *P*-value |
| --- | --- | --- | --- | --- | --- | --- | --- | --- | --- |
| Breast cancer resistance protein (%) | LS mean (SE) | 100.0 (2.1) | 100.7 (2.1) | 99.6 (2.1) | 101.8 (2.1) | 98.2 (2.1) | 96.7 (2.1) | 100.4 (2.1) | 0.7045 |
|  | LS mean difference (SE) |  | 0.7 (3.0) | –0.4 (3.0) | 1.8 (3.0) | –1.8 (3.0) | –3.3 (3.0) | 0.4 (3.0) |  |
|  | *P*-value |  | 0.9999 | 1.0000 | 0.9772 | 0.9787 | 0.7568 | 1.0000 |  |
| Multidrug resistance-associated protein 2 (%) | LS mean (SE) | 100.0 (2.2) | 103.0 (2.2) | 100.4 (2.2) | 101.9 (2.2) | 100.3 (2.2) | 95.1 (2.2) | 87.6 (2.2) | 0.0008 |
|  | LS mean difference (SE) |  | 3.0 (3.1) | 0.4 (3.1) | 1.9 (3.1) | 0.3 (3.1) | –4.9 (3.1) | –12.4 (3.1) |  |
|  | *P*-value |  | 0.8228 | 1.0000 | 0.9745 | 1.0000 | 0.4213 | 0.0031 |  |
| Bile salt export pump (%) | LS mean (SE) | 100.0 (24.2) | 166.9 (24.2) | 216.5 (24.2) | 220.3 (24.2) | 283.5 (24.2) | 178.5 (24.2) | 180.8 (24.2) | 0.0016 |
|  | LS mean difference (SE) |  | 66.9 (34.2) | 116.5 (34.2) | 120.3 (34.2) | 183.5 (34.2) | 78.5 (34.2) | 80.9 (34.2) |  |
|  | *P*-value |  | 0.2456 | 0.0131 | 0.0102 | 0.0001 | 0.1341 | 0.1180 |  |
| P-glycoprotein (%) | LS mean (SE) | 100.0 (6.2) | 104.5 (6.2) | 100.0 (6.2) | 104.8 (6.2) | 105.6 (6.2) | 95.3 (6.2) | 67.4 (6.2) | 0.0035 |
|  | LS mean difference (SE) |  | 4.5 (8.8) | –0.0 (8.8) | 4.8 (8.8) | 5.6 (8.8) | –4.7 (8.8) | –32.6 (8.8) |  |
|  | *P*-value |  | 0.9894 | 1.0000 | 0.9856 | 0.9679 | 0.9861 | 0.0064 |  |

LS means were estimated using an ANOVA including treatment groups as fixed factors. Pairwise comparison has been estimated using a Dunnett’s adjustment.

This table complements Supplementary Fig. S6.

EPL, essential phospholipids; LS, least-square; PI, phosphatidylinositol; PPC, polyenylphosphatidylcholine; SE, standard error

**
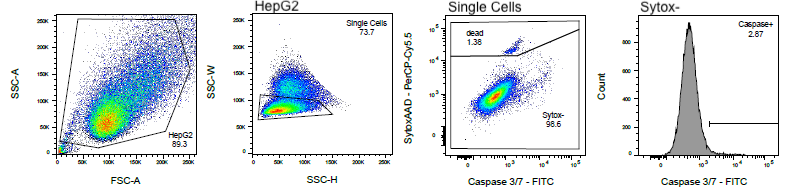
**

**Supplementary Fig. S1** Gating strategy to evaluate caspase-3/-7 activity in apoptotic cells by flow cytometry. Induction of apoptosis was measured by flow cytometric analysis of caspase-3/-7 activity. Cells were pregated by morphological features (size, granularity) to exclude cell debris and doublets. Caspase-3/-7 activity (FITC channel) was analysed in SytoxAAD (PerCP-Cy5.5 channel) negative cells to exclude dead cells. FC, flow cytometry; FITC, fluorescein isothiocyanate; FSC-A, forward scatter area; SSC-A, side scatter area; SSC-W, side scatter width; SSC-H, side scatter height


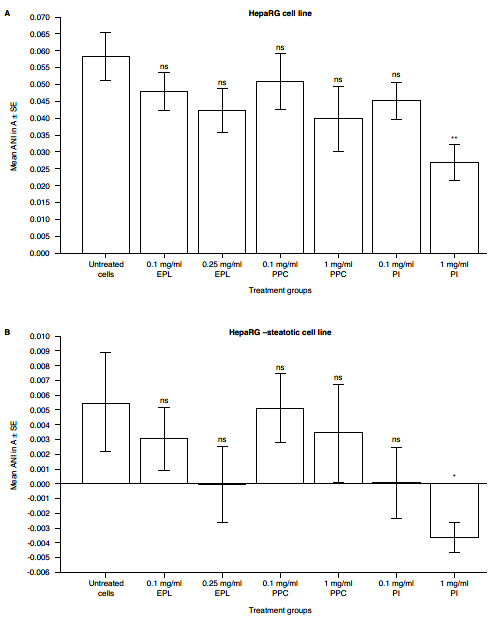


**Supplementary Fig. S2** Effect of EPL, PPC and PI on anisotropy in the HepaRG and steatotic HepaRG cell lines. Values shown are mean ± SE for 4 separate experiments; n = 6 replicates for each concentration of each compound per experiment (HepaRG); n = 3–6 replicates for each concentration of each compound per experiment (steatotic HepaRG). ns: not significant, **P* < 0.05, and ***P* < 0.01 versus untreated cells. ANI, anisotropy; EPL, essential phospholipids; PI, phosphatidylinositol; PPC, polyenylphosphatidylcholine; SE, standard error. Supplementary Table S2 shows the statistical analyses


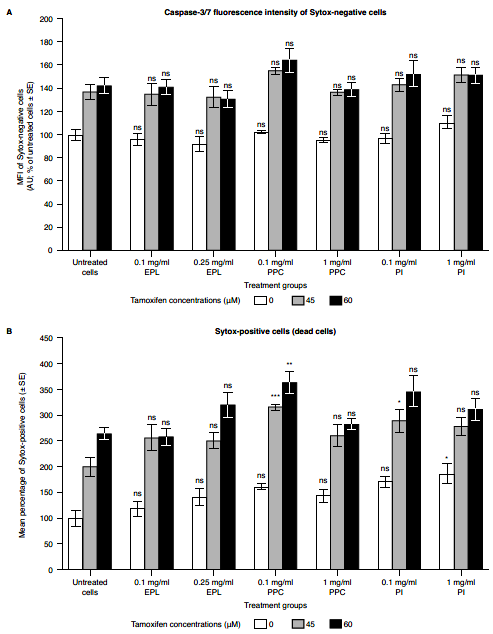


**Supplementary Fig. S3** Effect of EPL, PPC and PI on apoptosis in the HepaRG cell line. Values shown are mean ± SE (as % of untreated cells) for 2 separate experiments; n = 2 wells for each concentration of each compound per experiment. ns: not significant, **P* < 0.05, ***P* < 0.01, ****P* < 0.001 versus untreated cells. Note: for untreated HepaRG cells, 6.05% of cells were found to be Sytox positive (dead cells). Here, these values are presented as percentages, as the results are normalized to untreated cells. AU, arbitrary units; EPL, essential phospholipids; ns, not significant; MFI, median fluorescence intensity; PI, phosphatidylinositol; PPC, polyenylphosphatidylcholine; SE, standard error. Supplementary Table S4 shows the statistical analyses


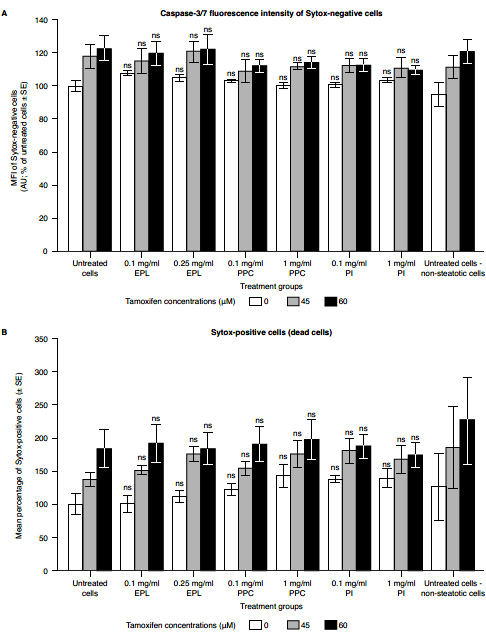


**Supplementary Fig. S4** Effect of EPL, PPC and PI on apoptosis in the steatotic HepaRG cell line. Values shown are mean ± SE (as % or untreated cells) for 2 separate experiments; n = 2 wells for each concentration of each compound per experiment. ns: not significant versus untreated cells. Note: for untreated steatotic HepRG cells, 4.55% of cells were found to be Sytox positive (dead cells). Here, these values are presented as percentages, as the results are normalized to untreated cells. AU, arbitrary units; EPL, essential phospholipids; ns, not significant; MFI, median fluorescence intensity; PI, phosphatidylinositol; PPC, polyenylphosphatidylcholine; SE, standard error. Supplementary Table S5 shows the statistical analyses


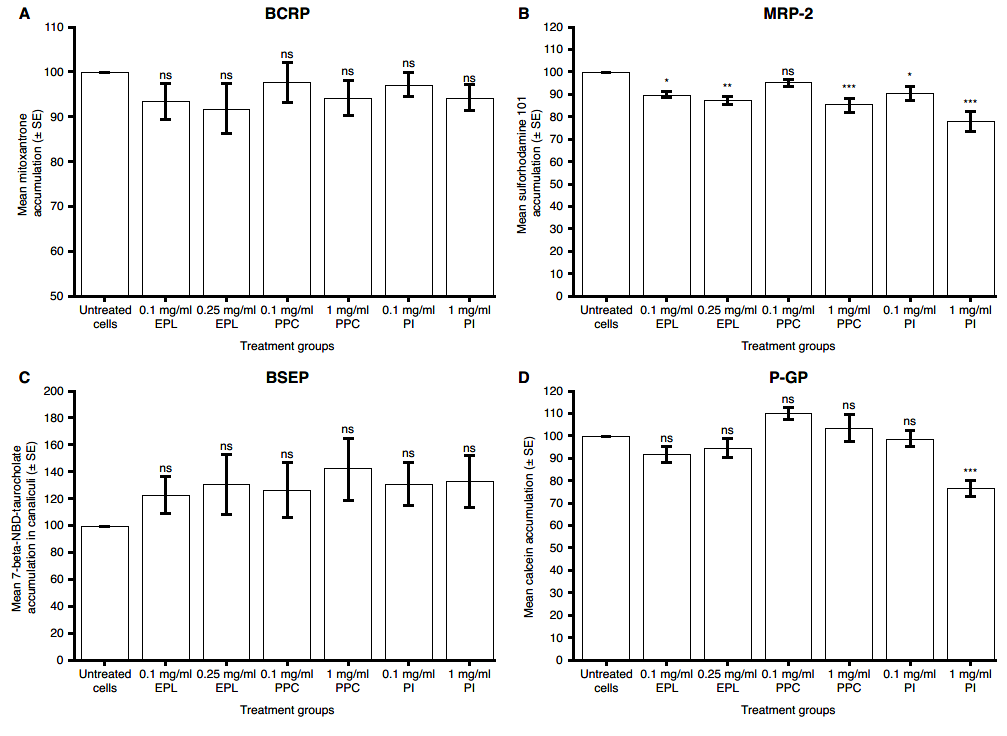


**Supplementary Fig. S5** Effect of EPL, PPC and PI on hepatocellular transport protein activity in the HepaRG cell line. Values shown are mean ± SE (substrate accumulation as percentage of untreated cells) for 4 experiments; n = 2 (BCRP), 2 or 3 (MRP-2, P-GP) or 4 (BSEP) wells for each concentration of each compound/experiment. ns = not significant, **P* < 0.05; ***P* < 0.01; ****P* < 0.001 versus untreated cells. BCRP, breast cancer resistance protein; BSEP, bile salt export protein; EPL, essential phospholipids; MRP-2, multidrug resistance-associated protein 2; NBD, 4-nitrobenzo-2-oxa-1,3-diazol; 2; P-GP, P-glycoprotein; PI, phosphatidylinositol; PPC, polyenylphosphatidylcholine; SE, standard error. Supplementary Table S7 shows the statistical analyses


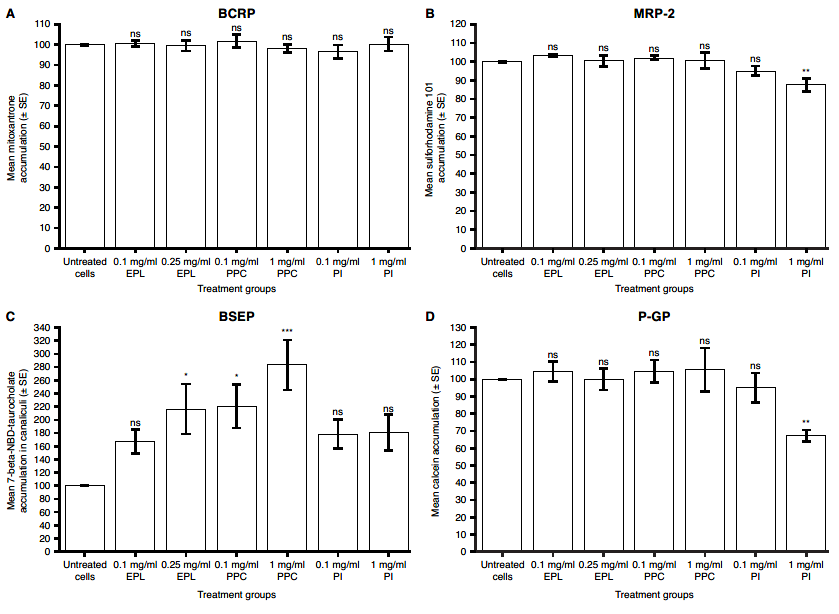


**Supplementary Fig. S6** Effect of EPL, PPC and PI on hepatocellular transport protein activity in the steatotic HepaRG cell line**.** Values shown are mean ± SE (substrate accumulation as percentage of untreated cells) for 4 experiments; n = 3 (BCRP), 4 (BSEP) or 6 (MRP-2, P-GP) wells for each concentration of each compound/experiment. ns = not significant, **P* < 0.05; ***P* < 0.01; ****P* < 0.001 versus untreated cells. BCRP, breast cancer resistance protein; BSEP, bile salt export protein; EPL, essential phospholipids; MRP-2, multidrug resistance-associated protein 2; NBD, 4-nitrobenzo-2-oxa-1,3-diazol; P-GP, P-glycoprotein; PI, phosphatidylinositol; PPC, polyenylphosphatidylcholine; SE, standard error. Supplementary Table S8 shows the statistical analyses
